# Metagenomics Unveils *Posidonia oceanica* “Banquettes” as a Potential Source of Novel Bioactive Compounds and Carbohydrate Active Enzymes (CAZymes)

**DOI:** 10.1128/mSystems.00866-21

**Published:** 2021-09-14

**Authors:** Esther Rubio-Portillo, Ana-Belen Martin-Cuadrado, Alfonso Ángel Ramos-Esplá, Josefa Antón

**Affiliations:** a Department of Physiology, Genetics and Microbiology, University of Alicante, Alicante, Spain; b Department of Marine Sciences and Applied Biology, University of Alicante, Alicante, Spain; c CIMAR, University of Alicante, Alicante, Spain; University of Technology Sydney

**Keywords:** BGC, CAZyme, *Myxococcota*, NRPS, *Posidonia oceanica*, banquettes, bioactive compound, *Pseudoalteromonas*

## Abstract

Posidonia oceanica is a long-living and very slow-growing marine seagrass endemic to the Mediterranean Sea. It produces large amounts of leaf material and rhizomes, which can reach the shore and build important banks known as “banquettes.” In recent years, interest in the potential uses of these *P. oceanica* banquettes has increased, and it was demonstrated that biomass extracts showed antioxidant, antifungal, and antiviral activities. The discovery of new compounds through the culture of microorganisms is limited, and to overcome this limitation, we performed a metagenomic study to investigate the microbial community associated with *P. oceanica* banquettes. Our results showed that the microbial community associated with *P. oceanica* banquettes was dominated by *Alphaproteobacteria*, *Gammaproteobacteria*, *Bacteroidetes*, and *Cyanobacteria*. *Pseudoalteromonas* was the dominant genus, followed by *Alteromonas*, *Labrenzia*, and *Aquimarina*. The metagenome reads were binned and assembled into 23 nearly complete metagenome-assembled genomes (MAGs), which belonged to new families of *Cyanobacteria*, *Myxococcota*, and *Granulosicoccaceae* and also to the novel genus recently described as *Gammaproteobacteria* family UBA10353. A comparative analysis with 60 published metagenomes from different environments, including seawater, marine biofilms, soils, corals, sponges, and hydrothermal vents, indicated that banquettes have numbers of natural products and carbohydrate active enzymes (CAZymes) similar to those found for soils and were only surpassed by marine biofilms. New proteins assigned to cellulosome modules and lignocellulose-degrading enzymes were also found. These results unveiled the diverse microbial composition of *P. oceanica* banquettes and determined that banquettes are a potential source of bioactive compounds and novel enzymes.

**IMPORTANCE** *Posidonia oceanica* is a long-living and very slow-growing marine seagrass endemic to the Mediterranean Sea that forms large amounts of leaf material and rhizomes, which can reach the shore and build important banks known as “banquettes.” These banquettes accumulate on the shore, where they can prevent erosion, although they also cause social concern due to their impact on beach use. Furthermore, *Posidonia* dry material has been considered a source of traditional remedies in several areas of the Mediterranean, and a few studies have been carried out to explore pharmacological activities of *Posidonia* extracts. The work presented here provides the first characterization of the microbiome associated with *Posidonia* banquettes. We carried out a metagenomic analysis together with an in-depth comparison of the banquette metagenome with 60 published metagenomes from different environments. This comparative analysis has unveiled the potential that *Posidonia* banquettes have for the synthesis of natural products, both in abundance (only surpassed by marine biofilms) and novelty. These products include mainly nonribosomal peptides and carbohydrate active enzymes. Thus, the interest of our work lies in the interest of *Posidonia* “waste” material as a source of new bioactive compounds and CAZymes.

## INTRODUCTION

Posidonia oceanica (L.) Delile is a long-living and very slow-growing marine seagrass, endemic to the Mediterranean Sea, that grows along the coast, forming extensive and highly productive meadows from shallow waters to depths up to 40 m (1–3). Meadows of *P. oceanica* and other seagrasses produce large amounts of leaf material that senesce and detach from the rhizomes from September to October. Detached leaf material may reach the shore, where it builds important banks known as “banquettes” (4). These banquettes of organic material help prevent coastal erosion caused by wave action (5, 6) and also provide a potential nutrient sink for the coastal trophic web (7, 8). Despite the benefits that these banquettes offer the shoreline, they cause great economic and social concern in coastal zones of the Mediterranean basin because they disturb beachgoers, necessitating banquette removal to the landfill at high cost (9).

In recent years, interest in the potential uses of these *P. oceanica* banquettes has increased. For instance, they have been tested as a fiber source for ruminants (10, 11) and have been used in agriculture as an organic substrate to increase soil fertility (12, 13). Moreover, *P. oceanica* has been used in traditional medicine to treat type 2 diabetes mellitus and hypertension in villagers living by the sea coast of Western Anatolia, as well as to alleviate skin diseases (i.e., acne) and leg pain caused by varicose veins (14). Indeed, *P. oceanica* ethanol extracts exerted antidiabetic and vasoprotective effects in rats (14). In addition, *P. oceanica* biomass extracts showed antioxidant capacity and antimicrobial activity against foodborne fungi and antiviral activity against human noroviruses (15).

Microbial communities associated with seagrass banquettes have been previously characterized by 16S rRNA gene sequencing and are dominated by facultative aerobes and copiotrophic groups, such as *Cellvibrionaceae*, *Rhodobacteracea*, and *Pseudoalteromas* species (16), which are able to form biofilms (17), and could be a good source of antimicrobial and bioactive compounds (reviewed in reference 18). Furthermore, as *P. oceanica* banquettes are composed of mainly cellulose, hemicellulose, and lignin (19), their associated microorganisms may decompose lignin and polysaccharides by encoding carbohydrate active enzymes (CAZymes) (20), which have diverse industrial and biotechnological applications in processing biomass into sugars, fuels, and other chemicals (21).

Among bioactive natural products, polyketide synthases (PKSs) and multienzymatic nonribosomal peptide synthetases (NRPSs) are of great interest to the pharmaceutical industry, because they can act as antibiotics, immunosuppressants, antitumor agents, toxins, or siderophores (22). Although most screenings for PKSs and NRPSs have been performed in soil samples (reviewed in reference 23), attention is turning to marine habitats that have great potential as a source of new natural products (24–26). Thus, the microbes and their encoded PKSs and NRPSs present in an underexplored marine habitat like *P. oceanica* banquettes are worth investigating as a source of new drugs and other natural products.

Discovery of novel substances typically involves culture-based approaches and screening crude extracts from natural sources. However, these methods fail to identify the majority of bioactive compounds produced by microorganisms because most secondary metabolic genes are “cryptic” and are not expressed under cultivation conditions (22, 26). Recent progress in full-genome sequence analysis of bacteria and fungi revealed that, for a given organism, there are far more biosynthetic gene clusters than currently known metabolites, suggesting that the biosynthetic potential for natural products in microorganisms has been greatly underexplored by traditional culture methods (27). Thus, approaches that do not rely on culture, such as metagenomics, are essential to not only estimate the microbial diversity in the environment but to also explore functional gene diversity and identify genes with biotechnological applications.

Here, we present the first metagenomic study of the microbial community associated with *P. oceanica* banquettes, unveiling its composition and potential as a source of bioactive compounds and novel enzymes. We also report the assembly of 23 nearly complete metagenome-assembled bacterial genomes (MAGs), which belong to newly discovered families of microbes. Together, these data provide the first detailed picture of microbial communities within *P. oceanica* banquettes, including their diversity and their degree of taxonomic novelty. Comparative analysis with 60 published metagenomes from different environments (seawater, marine biofilms, soils, corals, sponges, and hydrothermal vents) confirmed the great potential for banquettes as a natural product reservoir.

## RESULTS AND DISCUSSION

### Overall microbiome composition of *P. oceanica* banquettes.

Two approaches were used to investigate the biodiversity of the microbial community of *P. oceanica* banquettes, namely, 16S rRNA metabarcoding and metagenome shotgun sequencing.

**FIG 1:**
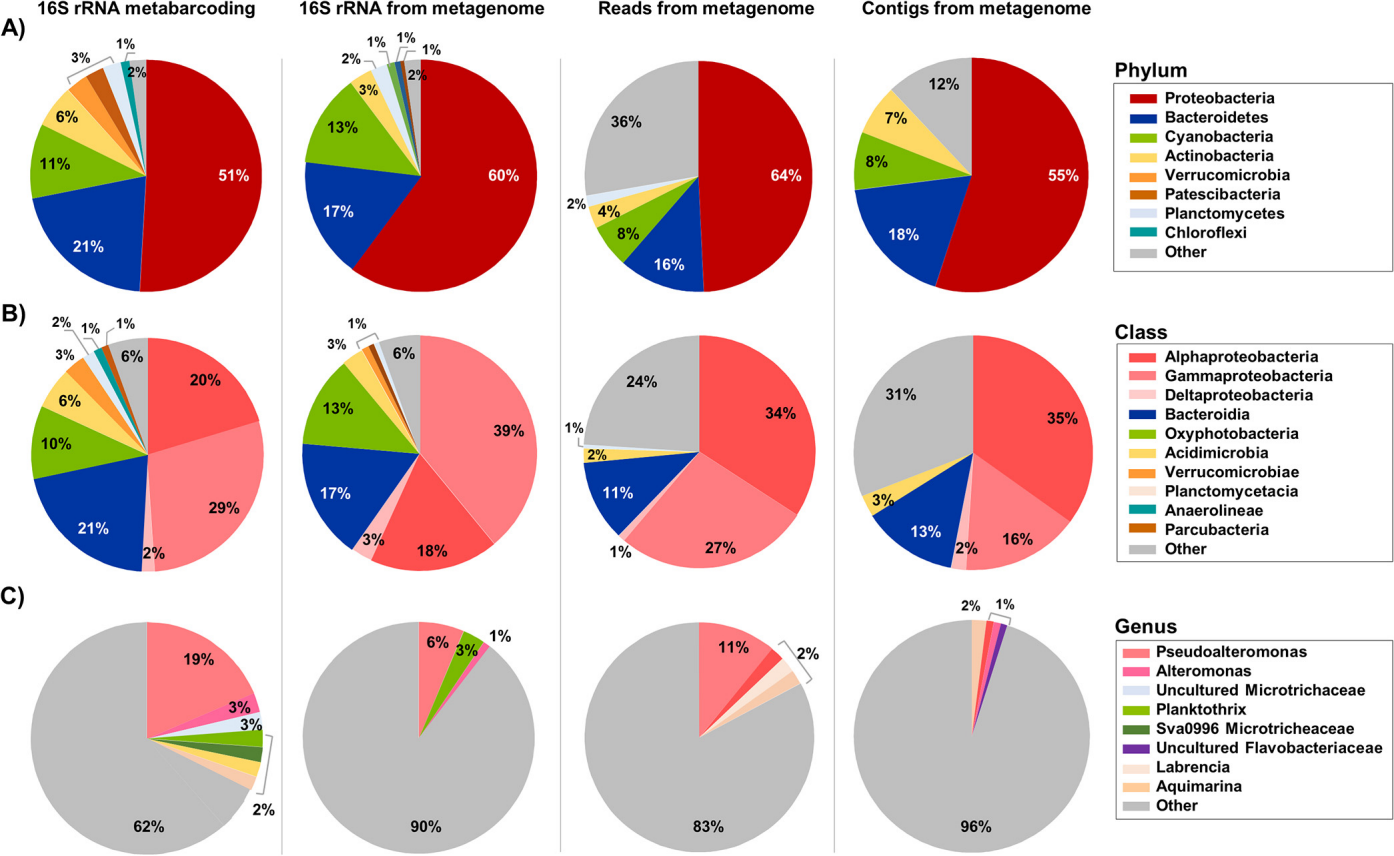
Taxonomic distribution of the microbial community recovered from *Posidonia oceanica* banquettes using different approaches (16S rRNA metabarcoding, 16S rRNA from metagenome shotgun sequencing, reads from metagenome shotgun sequencing, and contigs from metagenome shotgun sequencing) at the level of phylum (A), family (B), and genus (C).

**(i) Prokaryote taxonomy obtained by 16S rRNA gene metabarcoding.** A data set of 103,439 high-quality partial 16S rRNA gene sequences was generated after merging paired reads and excluding sequences of low quality or that were likely chimeric. Classification of 16S rRNA gene operational taxonomic units (OTUs) at 99% similarity resulted in a total of 20,277 OTUs. Microbes that dominated *P. oceanica* banquettes belonged to *Proteobacteria* (*Gammaproteobacteria*, 29%; *Alphaproteobacteria*, 23%), *Bacteroidetes*, and *Cyanobacteria*; microbes in these three phyla represented more than 80% of the sequences analyzed (Fig. 1A and B). *Pseudoalteromonas* was identified as the most prevalent genus (17.13%) associated with *P. oceanica* banquettes, followed by an uncultured *Microtrichaceae* genus (2.90%) and *Planktothrix* (2.80%) (Fig. 1C). The taxonomic profile from the 16S rRNA retrieved from metagenomic reads was congruent with the results using 16S rRNA metabarcoding (Fig. 1). This community was similar, at the phylum and class levels (82 to 95% and 80 to 91% Bray-Curtis similarity values, respectively), to those present in the other three samples collected at different beaches and sampling times. Overall, the same dominant genera were detected in all the samples although their relative abundances were different (see Fig. S8 in the supplemental material), and accordingly, Shannon indexes varied among samples (from 8.99 to 10.21). These results suggest that the main findings derived from the *P. oceanica* banquette metagenome shown below can be extrapolated to other samples.

10.1128/mSystems.00866-21.8 FIG S8 Taxonomic distribution of the microbial community recovered from *Posidonia oceanica* banquette samples, collected from San Juan beach in 2019 and from Rincón de la Zafra and Las Lanzas beaches in 2021, at the level of phylum (A), family (B), and genus (C). Download FIG S8, PDF file, 0.2 MB. Copyright © 2021 Rubio-Portillo et al. 2021 Rubio-Portillo et al. https://creativecommons.org/licenses/by/4.0/ This content is distributed under the terms of the Creative Commons Attribution 4.0 International license.

**(ii) Microbial taxonomy from metagenomic reads.** After quality filtering the initial 485,922,432 reads, a total of 477,184,531 reads remained. The coverage of metagenomic sequencing, assessed with Nonpareil (28), was 71.1%, which is an adequate sequencing depth to capture most of the microbial diversity in the banquettes. The average GC content was 50.6%, higher than the GC content reported in surface seawaters (for example, 38.6% in the Mediterranean Sea [29]) and lower than the GC content in soil samples (for example, 60% in tropical forest soils [30]). From all metagenomic sequences, analyzed with Kaiju, 23.90% were classified to the phylum level and 0.05% were classified as viruses (https://data.cyverse.org/dav-anon/iplant/home/amartin/Rubio-Portillo%20et%20al%202021%20-%20Supplementary%20Data/Krona%20plot%20from%20the%20Posidonia%20oceanica%20banquettes.html). The metagenomic sequence data set was dominated by *Bacteria* (99.38% of classified sequences), and surprisingly, even though *P. oceanica* is represented in the NCBI database, only 0.56% were classified as *Eukarya* (16.14% of them as *Fungi*). As the availability of genomes from eukaryotes is lower than for prokaryotes, it is possible that part of the 76% of the unclassified reads belonged to eukaryotic organisms. In order to clarify the presence of eukaryotes, these sequences were mapped against the small subunit (SSU) rRNA (SILVA_138_SSU) (31) database. The results showed that 35% of the detected rRNA originated from eukaryotes. Confirming the 16S rRNA metabarcoding results, *Proteobacteria* (*Alphaproteobacteria*, 34%, and *Gammaproteobacteria*, 27%), *Bacteroidetes*, and *Cyanobacteria* constituted 90% of the bacterial classified reads. *Pseudoalteromonas* was also confirmed as the dominant genus, accounting for 11% of the bacterial sequences, followed by *Alteromonas* (3.70%), *Labrenzia* (2.21%), and *Aquimarina* (2.04%), which were also detected by 16S rRNA metabarcoding at similar relative abundances (Fig. 1C).

**(iii) Taxonomy of the contigs assembled from the *P. oceanica* banquette metagenome.** The assembly process generated 6,094,075 contigs with a mean *N*_50_ of 910 bp. Contigs larger than 5 kb (*n* = 76,447) represented 25.47% of the nucleotides sequenced. These contigs mainly belonged to *Alphaproteobacteria* (35% of the total contigs), *Bacteroidetes* (18%), *Gammaproteobacteria* (16%), *Cyanobacteria* (8%), and *Actinobacteria* (7%) (Fig. 1A), resembling the results obtained by the metagenomic reads. Only 1.08% of the total contigs (833 contigs, 10.1 Mb) belonged to *Pseudoalteromonas* (sequencing depth, 71.8 ± 157×) (Data Set S1, tab 3), despite this genus being one of the dominant ones in the 16S rRNA data set, together with *Labrenzia* (1.80%), *Aquimarina* (1.37%), and *Alteromonas* (0.92%). Likely, the high genomic diversity within the *Pseudoalteromonas* population present in the *P. oceanica* banquettes prevented assembly of a high number of contigs (32). A total of 428 *Pseudoalteromonas* genomes were compared against the *P. oceanica* banquette data set, and a Pseudoalteromonas atlantica strain was the one most similar.

10.1128/mSystems.00866-21.10 DATA SET S1 Overall characteristics of MAGs recovered from *Posidonia oceanica* banquettes and the contigs used in this work. Tab 1, taxonomies of each MAG inferred using the GTDB-tk database and the MIGA platform. An asterisk indicates MAGs that were classified using phylogenomic reconstructions obtained with PhyloPhlan. Tab 2, main features of the contigs used in this work. Tab 3, average sequencing depth and taxonomic assignment of each assembled contig (<5 kb) from the *Posidonia oceanica* banquettes. Tab 4, classification of K and C domains using NaPDoS database in the metagenome-assembled genomes (MAGs) recovered from *Posidonia oceanica* banquettes. Download Data Set S1, XLSX file, 3.6 MB. Copyright © 2021 Rubio-Portillo et al. 2021 Rubio-Portillo et al. https://creativecommons.org/licenses/by/4.0/ This content is distributed under the terms of the Creative Commons Attribution 4.0 International license.

Although bacteria dominated the metagenomic data set, the most abundant contigs were classified as viruses. Among them, the most abundant contig (k141_4070505), accounting for 12.74% of viral reads, was a circular phage genome of 43 kb (43.77% of GC content) with a sequencing depth of 2,391× (Data Set S1, tab 3). Gene annotation indicated that this viral genome belonged to the Siphoviridae family, and its host was predicted to be a member of *Gammaproteobacteria*. This very abundant phage was compared to all known phages of *Pseudoalteromonas*, and very few similarities were detected (Fig. S9).

10.1128/mSystems.00866-21.9 FIG S9 Comparison of *Posidonia oceanica* contig K141-4070505 with closely related phage genomes. Download FIG S9, PDF file, 0.5 MB. Copyright © 2021 Rubio-Portillo et al. 2021 Rubio-Portillo et al. https://creativecommons.org/licenses/by/4.0/ This content is distributed under the terms of the Creative Commons Attribution 4.0 International license.

### Metagenome-assembled genomes in *P. oceanica* banquettes.

Binning of the assembled contigs from the banquette metagenome using Maxbin (33) resulted in 73 bins, whereas 108 bins were obtained using MetaBAT (34). Bins from each data set were then merged using DasTool (35), resulting in 44 bins, 23 of which met the quality thresholds to be considered MAGs (Table 1).

**TABLE 1:** Genomic features of the MAGs (completeness, >80%; contamination, <5%) assembled from the metagenome of *P. oceanica* banquettes^*a*^

MAG ID	Completeness (%)	Contamination (%)	Total length (Mbp)	No. of contigs (longest, in kb)	% GC content	CDS	16S rRNA, 23S rRNA, 5S rRNA^*b*^	No. of tRNAs	ANIr (>95%)	Relative abundance (nt/Mbp)
MAG.022	93.0	0.0	6.18	408 (118.063)	47.53	5,141	0, 1, 1	25	99.41	0.06
MAG.028	93.0	0.0	3.29	144 (109.491)	41.93	3,112	0, 0, 2	25	99.57	0.03
MAG.040	87.3	1.4	4.29	368 (57.707)	47.95	3,712	1, 0, 1	21	99.56	0.03
MAG.096	95.8	4.2	10.16	644 (81.137)	44.48	8,046	2, 0, 1	32	99.51	0.08
MAG.080	83.1	1.4	2.20	146 (55.985)	42.32	2,133	1, 0, 0	26	99.49	0.02
MAG.050	91.5	1.4	5.43	461 (54.582)	34.12	4,166	0, 1, 1	27	99.77	0.04
MAG.088	94.4	4.2	5.11	377 (67.535)	46.72	4,579	0, 0, 0	35	99.49	0.04
MAG.114	83.1	1.4	2.48	161 (65.553)	47.96	2,369	0, 1, 0	29	99.83	0.02
MAG.005	90.1	2.8	9.82	254 (179.540)	69.34	8,436	0, 0, 2	58	99.76	0.12
MAG.019	97.2	0.0	5.39	257 (159.316)	52.18	4,257	0, 1, 1	35	99.57	0.15
MAG.121	85.9	1.4	0.64	3 (379.352)	46.19	717	2, 3, 1	35	99.86	0.01
MAG.069	90.1	2.8	3.62	246 (59.057)	62.00	3,275	1, 1, 1	35	99.65	0.03
MAG.004	85.9	1.4	2.79	255 (37.221)	56.66	2,759	0, 0, 0	20	99.71	0.02
MAG.057	84.5	1.4	3.90	326 (68.784)	61.31	3,779	1, 0, 0	35	99.48	0.03
MAG.058	94.4	0.0	4.28	15 (896.562)	51.64	3,822	0, 0, 2	53	99.82	0.14
MAG.066	97.2	0.0	1.87	87 (74.932)	46.55	1,094	1, 1, 1	30	99.78	0.02
MAG.036	81.7	2.8	3.78	382 (53.586)	54.18	3,504	0, 0, 0	18	99.14	0.03
MAG.054	93	1.4	5.26	360 (107.682)	48.85	5,001	0, 1, 2	21	99.39	0.05
MAG.106	87.3	0.0	9.61	766 (94.571)	50.33	8,899	0, 0, 2	49	99.31	0.11
MAG.079	87.3	1.4	3.27	270 (78.019)	43.49	3,474	0, 1, 1	28	99.79	0.11
MAG.122	88.7	4.2	3.72	150 (120.257)	48.09	3,447	0, 0, 0	35	99.58	0.04
MAG.082	81.7	4.2	3.39	370 (56.496)	49.14	2,643	0, 0, 0	19	99.03	0.05
MAG.032	98.6	4.2	3.53	198 (126.550)	49.04	3,254	0, 0, 0	35	99.82	0.04

a The relative abundance of each MAG was estimated using fragment recruitment analyses carried out by BLASTn comparisons. Only reads that matched with over 95% identity and 70% coverage were considered. The fraction of nucleotides mapping to the respective MAG was normalized by the length of that MAG and size of the metagenome. CDS, coding DNA sequence.

b Copy number of each of the three genes, respectively.

Microbial populations represented by MAGs had very low relative abundances and high average nucleotide identity of mapped read (ANIr) values (>99%) (Table 1), higher than the species-level cutoff of 95% (36). Thus, MAGs recovered here represent rare biosphere members with low microdiversity, as has been documented previously (32).

MAGs were taxonomically classified as *Proteobacteria* (11 MAGs), *Bacteroidetes* (6 MAGs), *Cyanobacteria* (2 MAGs), *Myxococcota* (2 MAGs), *Patescibacteria* (1 MAG), and *Planctomycetota* (1 MAG). Most of the MAGs could represent novel bacterial taxa based on their average amino acid identity (AAI) and ANI values (Table 2; Fig. S1 to S7). Four MAGs belonged to related bacterial taxa previously reported as microbiota of the seagrass (37, 38); MAG036, MAG054, and MAG106 were classified as *Granulosicoccus* species, and MAG069 was classified as *Planctomycetes*-related bacteria, reported as the dominant phylum in biofilms of *P. oceanica* leaves (39, 40).

**TABLE 2:** Taxonomic affiliations of the MAGs assembled from the metagenome of *P. oceanica* banquettes

Taxonomy				GTDB-tk closest neighbor match	AAI^*a*^	MAG ID
*Bacteroidota*	*Rhodothermia*	*Rhodothermales*		*Rhodothermaceae* bacterium (CP020382)	52.11	MAG.022
	*Bacteroidia*	*Flavobacterales*	*Flavobacteraceae*	*Aureitalea* sp. (CP027062)	64.61	MAG.028
		*Chinophagales*	*Saprospiraceae*	*Phaeodactylibacter* sp. (GCA_011374895.1)	54.78	MAG.040
		*Chinophagales*	*Saprospiraceae*	*Maribacter* sp. (NC 014472)	45.69	MAG.096
		*Chinophagales*	*Saprospiraceae*	*Saprospiraceae* bacterium (GCA_011374565.1)	52.49	MAG.080
		*Cytophagales*	*Flammeovirgaceae*	Fabibacter pacificus (*Flammeovigaceae*)	50.15	MAG.050
*Cyanobacteria*	*Cyanobacteriia*	*Phormidesmiales*	*Phormidesmiaceae*	*Leptolyngbya valderiana* (GCA_001637395.1)	56.88	MAG.088
	*Vampirovibrionia*	*Vampirovibrionales*	*Vampirovibrionaceae*	“*Candidatus* Melainabacteria bacterium” (GCF_902168245)	57.01*	MAG.114
*Myxococcota*	UBA796	UBA796	GCA-2862545	*Myxococcales*_bacterium (GCA_009692585)	49.08*	MAG.005
	UBA727			*Corallococcus exercitus* strain AB043A (GCA_003611585)	46.69*	MAG.019
*Patescibacteria*	*Paceibacteria*	UBA9983_A	UBA2163	“*Candidatus* Kaiserbacteria” (GCA_002773395)	64.92	MAG.121
*Planctomycetota*	*Phycisphaerae*	*Phycispharales*	*Phycispharaceae*	Phycisphaera mikurensis (GCA_002686995.1)	52.90	MAG.069
*Proteobacteria*	*Alphaproteobacteria*	*Rhizobiales*	*Rhodomicrobiaceae*	*Shinella* sp. (CP015736)	49.30	MAG.004
		*Rhodobacterales*	*Rhodobacteraceae*	*Rhodobacteraceae* bacterium (CP040818)	53.38	MAG.057
			*Thalassospiraceae*	Labrenzia aggregata (GCF_001999245.1)	54.04	MAG.058
		*Micavibrionales*	*Micavirbionaceae*	*Micavibrio* sp. (GCA_002687495)	67.51	MAG.066
	*Gammaproteobacteria*	*Grannulosicoccales*	*Granulosicoccaceae*	Granulosicoccus antarcticus (GCF_002215215.1)	59.20**	MAG.036
				Granulosicoccus antarcticus (GCF_002215215.1)	61.24**	MAG.054
				Granulosicoccus antarcticus (GCF_002215215.1)	63.86***	MAG.106
		UBA10353	UBA7415	*Cycloclasticus* sp. (GCA_002101205.1, symbiont of Poecilosclerida)	52.89**	MAG.079
			LS-SOB	*Acidiferrobacter* sp. (GCA_002694065)	54.12**	MAG.122
		*Pseudomonadales*	*Cellvibrionaceae*	Teredinibacter turnerae (NC 012997)	57.04	MAG.082
				Teredinibacter turnerae (NC 012997)	53.85	MAG.032

a Based on phylogenomic reconstructions, new families (*), new genera (**), and new species (***) are indicated.

10.1128/mSystems.00866-21.1 FIG S1 Phylogenomic assignment of assembled genome bins MAG19 and MAG005. The phylogenomic tree was obtained with PhyloPhlAn using broadly conserved proteins to extract phylogenomic signal. Organisms are colored based on genome size (Mb, in brackets). Download FIG S1, PDF file, 0.1 MB. Copyright © 2021 Rubio-Portillo et al. 2021 Rubio-Portillo et al. https://creativecommons.org/licenses/by/4.0/ This content is distributed under the terms of the Creative Commons Attribution 4.0 International license.

10.1128/mSystems.00866-21.2 FIG S2 Phylogenomic assignment of assembled genome bins MAG069. The phylogenomic tree was obtained with PhyloPhlAn using broadly conserved proteins to extract phylogenomic signal. Download FIG S2, PDF file, 0.1 MB. Copyright © 2021 Rubio-Portillo et al. 2021 Rubio-Portillo et al. https://creativecommons.org/licenses/by/4.0/ This content is distributed under the terms of the Creative Commons Attribution 4.0 International license.

10.1128/mSystems.00866-21.3 FIG S3 Phylogenomic assignment of assembled genome bins MAG122 and MAG079. The phylogenomic tree was obtained with PhyloPhlAn using broadly conserved proteins to extract phylogenomic signal. Download FIG S3, PDF file, 0.2 MB. Copyright © 2021 Rubio-Portillo et al. 2021 Rubio-Portillo et al. https://creativecommons.org/licenses/by/4.0/ This content is distributed under the terms of the Creative Commons Attribution 4.0 International license.

10.1128/mSystems.00866-21.4 FIG S4 Phylogenomic assignment of assembled genome bin MAG121. The phylogenomic tree was obtained with PhyloPhlAn using broadly conserved proteins to extract phylogenomic signal. Download FIG S4, PDF file, 0.1 MB. Copyright © 2021 Rubio-Portillo et al. 2021 Rubio-Portillo et al. https://creativecommons.org/licenses/by/4.0/ This content is distributed under the terms of the Creative Commons Attribution 4.0 International license.

10.1128/mSystems.00866-21.5 FIG S5 Phylogenomic assignment of assembled genome bin MAG066. The phylogenomic tree was obtained with PhyloPhlAn using broadly conserved proteins to extract phylogenomic signal. Download FIG S5, PDF file, 0.1 MB. Copyright © 2021 Rubio-Portillo et al. 2021 Rubio-Portillo et al. https://creativecommons.org/licenses/by/4.0/ This content is distributed under the terms of the Creative Commons Attribution 4.0 International license.

10.1128/mSystems.00866-21.6 FIG S6 Phylogenomic assignment of assembled genome bin MAG114. The phylogenomic tree was obtained with PhyloPhlAn using broadly conserved proteins to extract phylogenomic signal. Download FIG S6, PDF file, 0.1 MB. Copyright © 2021 Rubio-Portillo et al. 2021 Rubio-Portillo et al. https://creativecommons.org/licenses/by/4.0/ This content is distributed under the terms of the Creative Commons Attribution 4.0 International license.

10.1128/mSystems.00866-21.7 FIG S7 Phylogenomic assignment of assembled genomes MAG036, MAG054, and MAG106. The phylogenomic tree was obtained with PhyloPhlAn using broadly conserved proteins to extract phylogenomic signal. Download FIG S7, PDF file, 0.1 MB. Copyright © 2021 Rubio-Portillo et al. 2021 Rubio-Portillo et al. https://creativecommons.org/licenses/by/4.0/ This content is distributed under the terms of the Creative Commons Attribution 4.0 International license.

Notable among the recovered MAGs was the presence of heterotrophic bacteria with the ability to use complex carbon sources, along with species associated with predation of other bacteria and algae; i.e., MAG040, MAG080, and MAG096 that belonged to the *Saprospiraceae* family, whose members are known for degrading chitin, pectin, and cellulose (41). Members of this family are found in aquatic environments (marine and freshwater) and activated sludges. MAG032 and MAG082 were related to the *Teredinibacter* genus, which are copiotrophic marine bacteria with the ability to utilize complex polysaccharides as substrates (42).

*Myxococcota* species, with which MAG005 and MAG019 were most closely related, are commonly found in soil environments but have also been found in the seagrass rhizosphere (17). In general, *Myxococcota* possess large genomes, up to 16 Mb (43), and some of them can form aggregates in which extracellular enzymes and secondary metabolites have accumulated or develop fruiting bodies when nutrients are scarce (43). Although we specifically explored the presence of genes related to movement, predation, lysis, fruiting body formation, and sporulation in these MAGs, no differences were found in the numbers of genes for each of these categories among MAG005 and MAG019, in spite of their different sizes. One must keep in mind that genes related to fruiting body formation and predation may be harbored in the accessory genome and then, by the very nature of MAGs (44), may have not been binned with the core genome.

In summary, the four approaches (16S rRNA metabarcoding, read taxonomy, contig taxonomy, and MAGs) showed that the microbial community associated with *P. oceanica* banquettes was a mixture of members of the *P. oceanica* phyllosphere microbiome, dominated by *Planctomycetes* (39), together with typical marine microbes like *Alphaproteobacteria*, *Bacteroidia*, and *Oxyphotobacteria* (29) and common soil bacterial taxa such as *Actinobacteria*, *Acidobacteria*, or *Verrucomicrobia* (45). This suggests the probable influence of both seawater and beach sand in structuring this ecosystem. Moreover, the *P. oceanica* banquette microbiome was dominated by *Pseudoalteromonas* spp., which is a common genus in copiotrophic communities during short-term seagrass decomposition (16). *Pseudoalteromonas* species are also typical epibiotic bacteria in marine photosynthetic organisms, including macroalgae and seaweeds, which are able to produce antibacterial products and polymer-degrading enzymes (46–48), as well as a wide range of enzymes that may assist it in competition for nutrients and space and in protection against grazing predators (49). These characteristics, together with ligninolytic and chitinolytic activity that has been reported in some *Pseudoalteromonas* species (50, 51), suggest that this genus is effective in colonizing and degrading *P. oceanica* beach banquettes.

### Secondary metabolite production.

**FIG 2:**
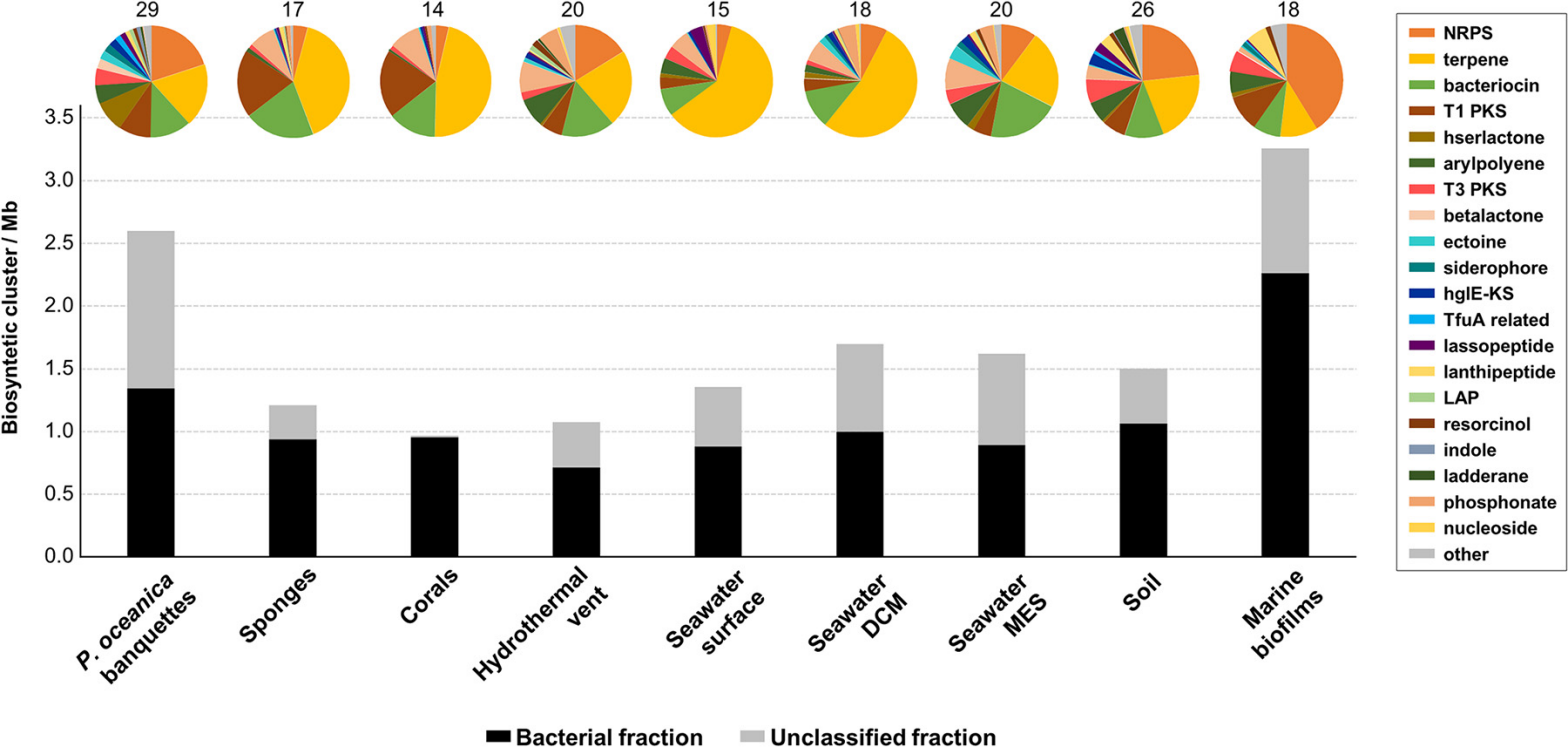
Relative frequencies of the biosynthetic clusters detected in *Posidonia oceanica* banquettes and other environments using antiSMASH (in contigs of >5 kb). Black bars indicate the numbers of clusters detected among the bacterial contigs, and gray bars indicate those unclassified taxonomically. Pie charts above the bars indicate the relative contribution of each biosynthetic cluster category according to the legend. The number above each pie chart represents the number of different biosynthetic clusters. DCM, deep chlorophyll maximum; MES, mesopelagic; LAP, linear azol(in)e-containing peptide.

Since *P. oceanica* dry material has been considered a source of traditional medicine remedies and their extracts showed antimicrobial and antiviral activities, we have carried out a secondary biosynthetic cluster search in the banquette-associated microorganisms. For this purpose, contigs over 5 kb were used to determine the number of secondary metabolite biosynthetic gene clusters (BGCs). To compare the metabolic potential of *P. oceanica* banquettes with that of other well-known environments, a total of 60 metagenomic contig collections (>5 kb) from different environments (Data Set S1, tab 2) were also analyzed using AntiSMASH. Remarkably, metagenomes from the *P. oceanica* banquettes and marine biofilms had more BGCs than those from soil, which has been considered an environment linked to secondary metabolite production (52) (Fig. 2). Compared with other biomes, the *P. oceanica* banquette microbiome had the largest number of BGCs among the unclassified contigs (1.66-fold more than in soil samples), suggesting that a great proportion of these clusters are in underexplored microbes. Furthermore, *P. oceanica* banquette BGCs were also the most diverse (Fig. 2).

Polyketide synthases (PKSs) and nonribosomal peptide synthetases (NRPSs) are encoded by two families of BGCs that are of great interest to the biotechnological industry, since they are used in the production of antibiotics, antitumor agents, and immunosuppressants, among other compounds (53, 54). NRPSs have a modular structure with multiple domains, including the condensation (C) domain, which is a key component that forms a peptide bond between the next amino acyl and the peptidyl unit. Modifying domains for epimerization, heterocyclization, or oxidation could be additionally integrated. On the other hand, PKSs are assembled from acyl units. Selection of the monomers is performed by acyltransferase (AT) domains, and ketoacyl synthase (KS) domains are responsible for the elongation step.

**FIG 3:**
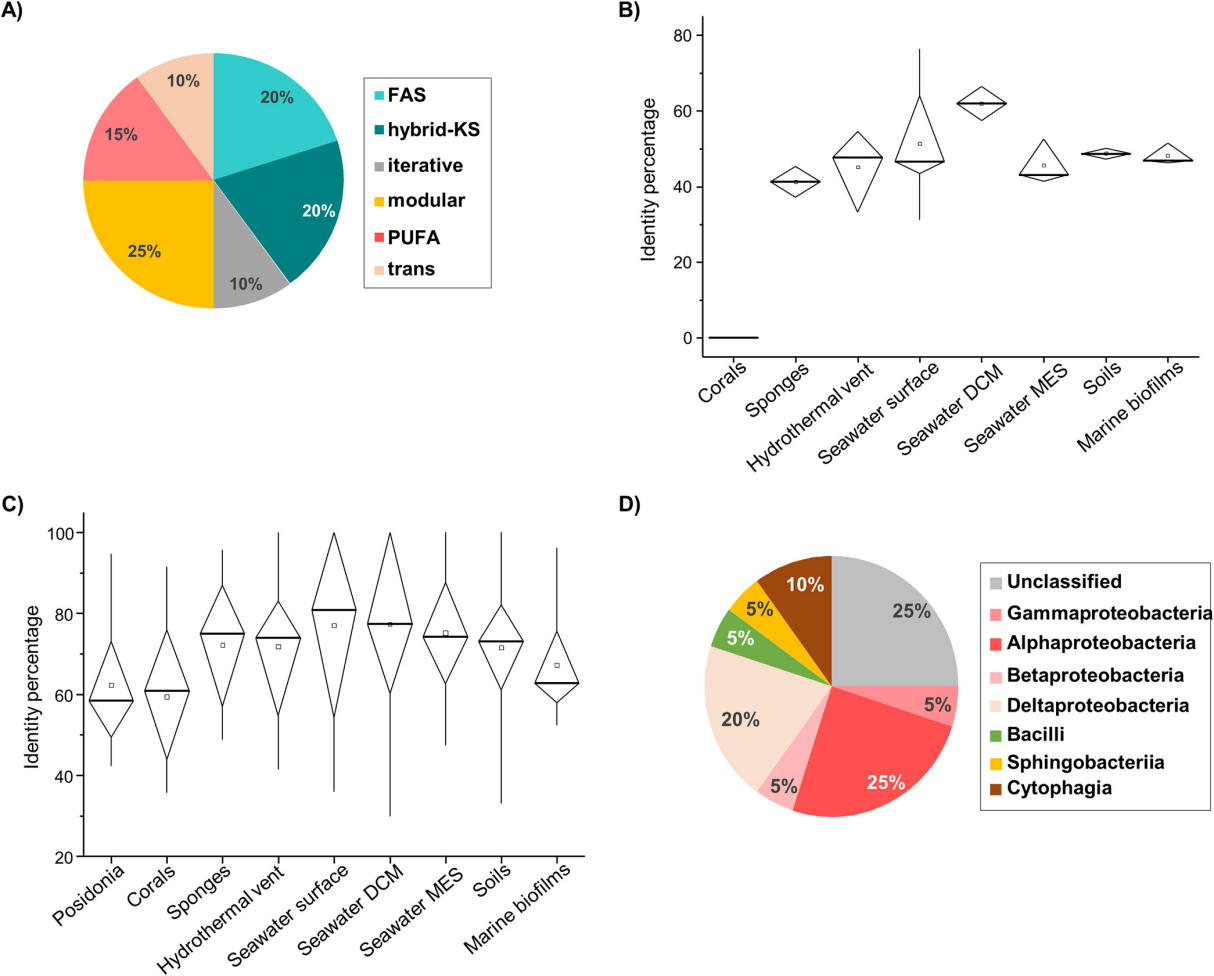
Ketoacyl synthase (KS) domains in polyketide synthases (PKSs) detected in *Posidonia oceanica* banquettes. (A) Classification of KS domains. (B) Box plot of similarities between the KS domains detected in *P. oceanica* and those found in the other environments analyzed in this work. (C) Box plot of similarities between the KS domains detected in each habitat used in this work against proteins in the nr database. (D) Taxonomic distribution of KS domains detected in *P. oceanica* banquettes. FAS, domains involved in fatty acid synthesis; hybrid-KS, biosynthetic assembly lines that include both PKS and NRPS components; iterative, PKS domains containing the characteristic domains of type I PKSs; modular, multidomain architecture consisting of multiple sets of modules; PUFA, polyunsaturated fatty acid; trans, modular PKS operons lacking cognate AT domains; DCM, deep chlorophyll maximum; MES, mesopelagic.

A total of 158 sequences from *P. oceanica* banquettes were predicted to have a KS domain. These sequences grouped into 21 different clusters (cutoff, 40% similarity) that belonged to 6 different KS classes (Fig. 3A). The relative abundance of KS domains present in *P. oceanica* banquettes was lower than that in other environments studied here and showed highest similarity with those found in the deep chlorophyll maximum (DCM) and surface seawaters (Fig. 3B). Comparison of PKSs detected in *P. oceanica* banquettes with sequences in the nonredundant (nr) database showed high sequence similarity (Fig. 3C); however, three KS domains were found to be unique, with no similar sequences in the other environments or in the nr database. Among the identified PKS domains, the majority of the contigs belonged to the phylum *Proteobacteria* (55%), mainly to the classes *Alphaproteobacteria* and Deltaproteobacteria (Fig. 3D).

**FIG 4:**
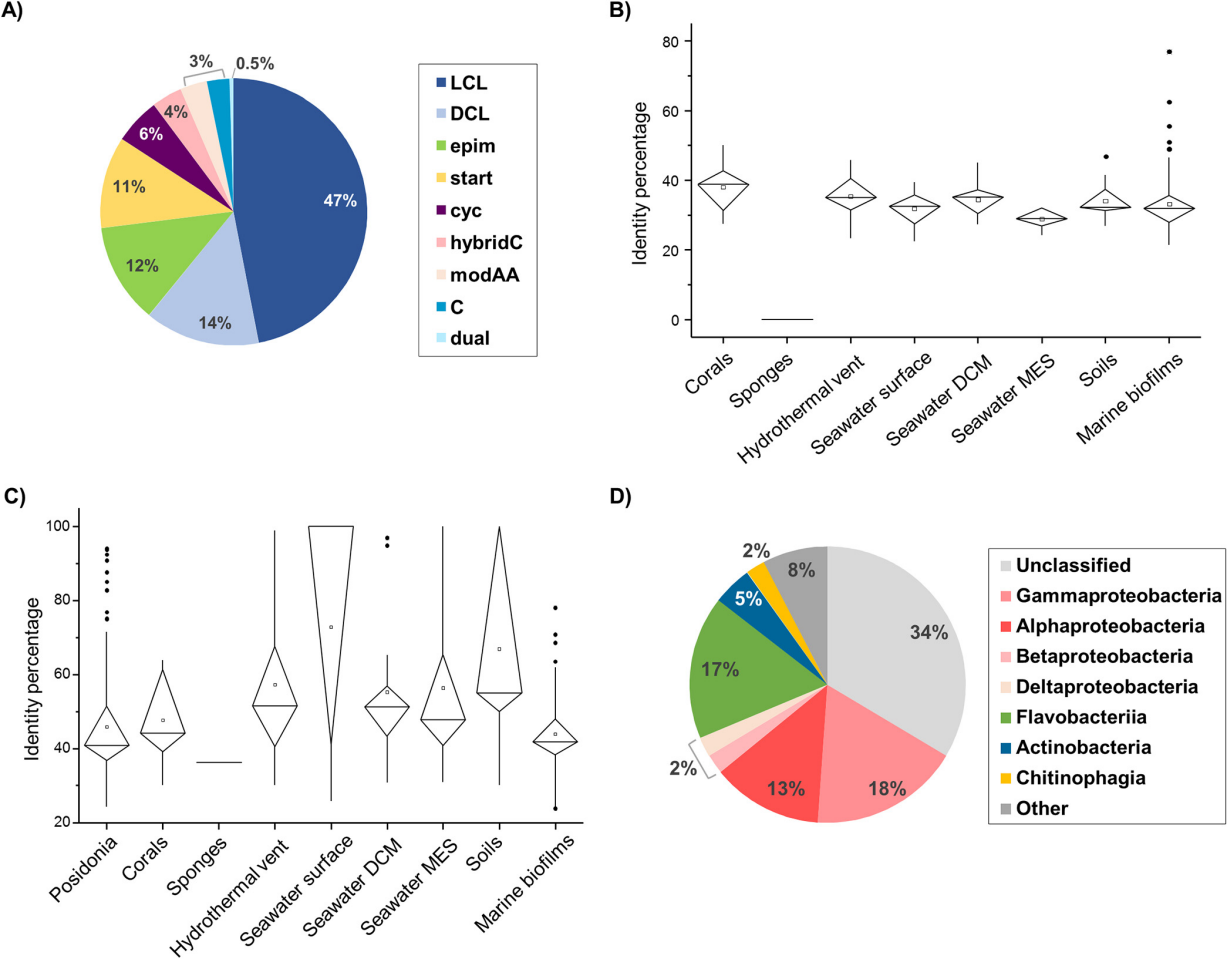
Condensation domains in nonribosomal peptide synthetases (NRPSs) detected in *Posidonia oceanica* banquette contigs (>5 kb). (A) Classification of C domains. (B) Box plot of similarities between the C domains from *P. oceanica* banquettes and those detected in other environments. (C) Box plot of similarities between the C domains from the different environments and proteins in the nr database. (D) Taxonomic distribution of C domains detected in *P. oceanica* banquettes. C, condensation domain; cyc, cyclization domains; DCL, domains that link an l-amino acid to a growing peptide ending with a d-amino acid; dual, dual domains catalyzing condensation and epimerization; epim, epimerization domains changing the chirality of the last amino acid in the chain from l- to d-amino acid; hybridC, hybrid PKS/NRPS secondary metabolite; LCL, domains that catalyze formation of a peptide bond between two l-amino acids; modAA, involved in the modification of the incorporated amino acid; start, acylate the first amino acid with a fatty acid, polyketide, or other molecule; DCM, deep chlorophyll maximum; MES, mesopelagic.

A total of 391 C domains (NRPSs) were detected among the contigs that were clustered into 216 groups; thus, the diversity of NRPSs was much higher than that of PKSs in *P. oceanica* banquettes. (Fig. 4A). The relative abundance of NRPSs in *P. oceanica* was similar to that in soil samples and higher than that in seawater, but it was found to be lower than that in marine biofilms (Fig. 2). Only 35 of the 216 representative C domain clusters detected in *P. oceanica* banquettes showed more than 40% similarity with C domain containing sequences from other environments (Fig. 4B). Similarities were also limited when metagenomic sequences were compared against sequences in the nr database (Fig. 4C). Indeed, 181 of the C domains recovered from *P. oceanica* banquettes were not found among the other environments analyzed, and 90 of them lacked homologues in the nr database, indicating that these were novel NRPSs. Inference of the taxonomic origins of the contigs encoding the C domains in *P. oceanica* banquettes showed that these contigs belonged to 11 different phyla. *Proteobacteria*, mainly *Gammaproteobacteria* and *Alphaproteobacteria* classes, together with *Bacteroidetes* and *Flavobacteria* classes, represented almost 50% of the contigs with NRPS domains, while 33% of the contigs were not classified at the class level (Fig. 4D). Among the genera that carried C domains in the *P. oceanica* banquettes, *Marinomonas*, *Aquimarina*, and *Leptolyngnya* were predicted to be the main NRPS producers.

**FIG 5:**
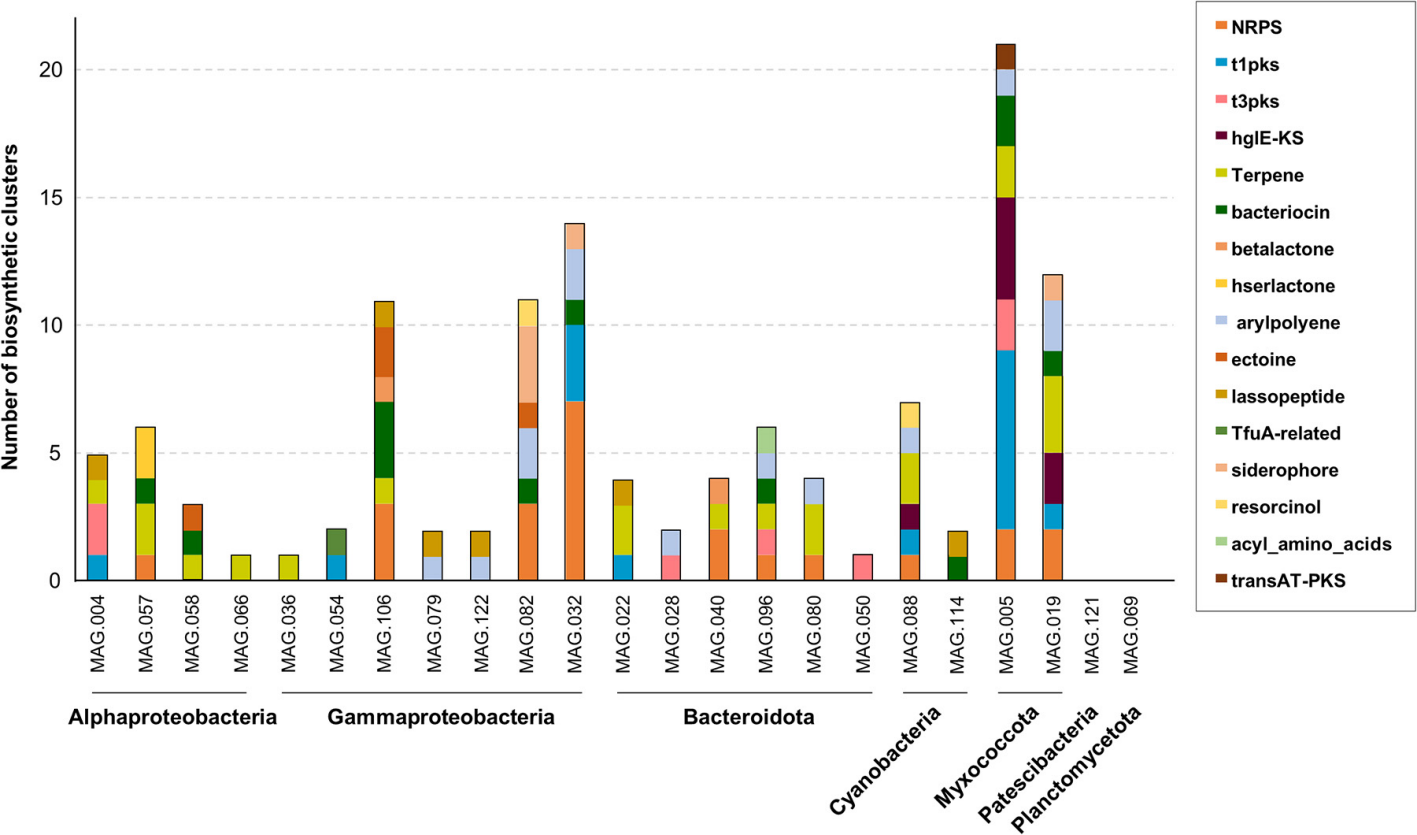
Classification of secondary metabolite biosynthetic gene clusters in the 23 metagenome-assembled genomes (MAGs) recovered from *Posidonia oceanica* banquettes.

The presence of BGCs in the MAGs recovered from *P. oceanica* banquettes was also investigated (Fig. 5). Although these MAGs did not correspond to dominant microbes in the sample, they could be useful for designing future BGC recovery strategies. Of the 23 recovered MAGS, only two (belonging to the *Patescibacteria* and *Planctomycetota* phyla) lacked BGCs. Five MAGs (MAG005, MAG019, MAG032, MAG082, and MAG106) harbored more than 10 BGCs. Among them, MAG005 and MAG019 showed the greatest proportion of NRPS or PKS clusters; e.g., MAG005 had 2 NRPS clusters, 9 PKS clusters, and 4 heterocyst glycolipid synthase-like PKSs (HgIE-KS) (Fig. 5). The KS and C domains detected were putative modular I PKSs, similar to those found in the pathway for epothilone synthesis (Data Set S1, tab 4). Epothilones are potential cancer drugs which act as tubulin polymerization agents (55). In MAG005, three of the HgIE-KS domains were related to polyunsaturated fatty acid production; however, the two NRPS domains could not be identified (Data Set S1, tab 4). As mentioned above, MAG005 and MAG019 belonged to the *Myxococcota* phylum, which, together with *Actinobacteria*, is well known for its ability to produce natural compounds, mainly in soil ecosystems (56). However, these two MAGs clustered with marine myxobacteria that, although underrepresented in comparison to their soil counterparts (57), are also known to produce interesting bioactive compounds (58).

MAG032 and MAG082, which were classified as belonging to the *Teredinibacter* genus, were also predicted to be important NRPS and PKS producers (Fig. 5). Teredinibacter turnerae has been described as a cellulolytic/nitrogen-fixing bacterium found as part of the microbial endosymbiotic consortium that supports the wood-boring lifestyle of mollusks (59). This microbe displays antimicrobial activities against Gram-negative and Gram-positive bacteria (60), and its potential as a secondary metabolite producer is similar to that of some *Actinobacteria* (61). Five of the NRPS domains identified among MAG032 and MAG082 were classified as C domains related to the yersiniabactin and bleomycin pathways. In MAG032, three PKS domains were classified as trans-AT PKS from the leinamycin pathway (a macrolactam with antitumor and antimicrobial properties [62]), and also identified were three hybrid PKS/NRPS domains that could belong to the yersiniabactin and epothilone pathways (Data Set S1, tab 4). Overall, these results indicate that *P. oceanica* banquettes harbor novel *Myxococcota* and *Teredinibacter* species with a high biosynthetic potential.

### CAZyme analysis.

**FIG 6:**
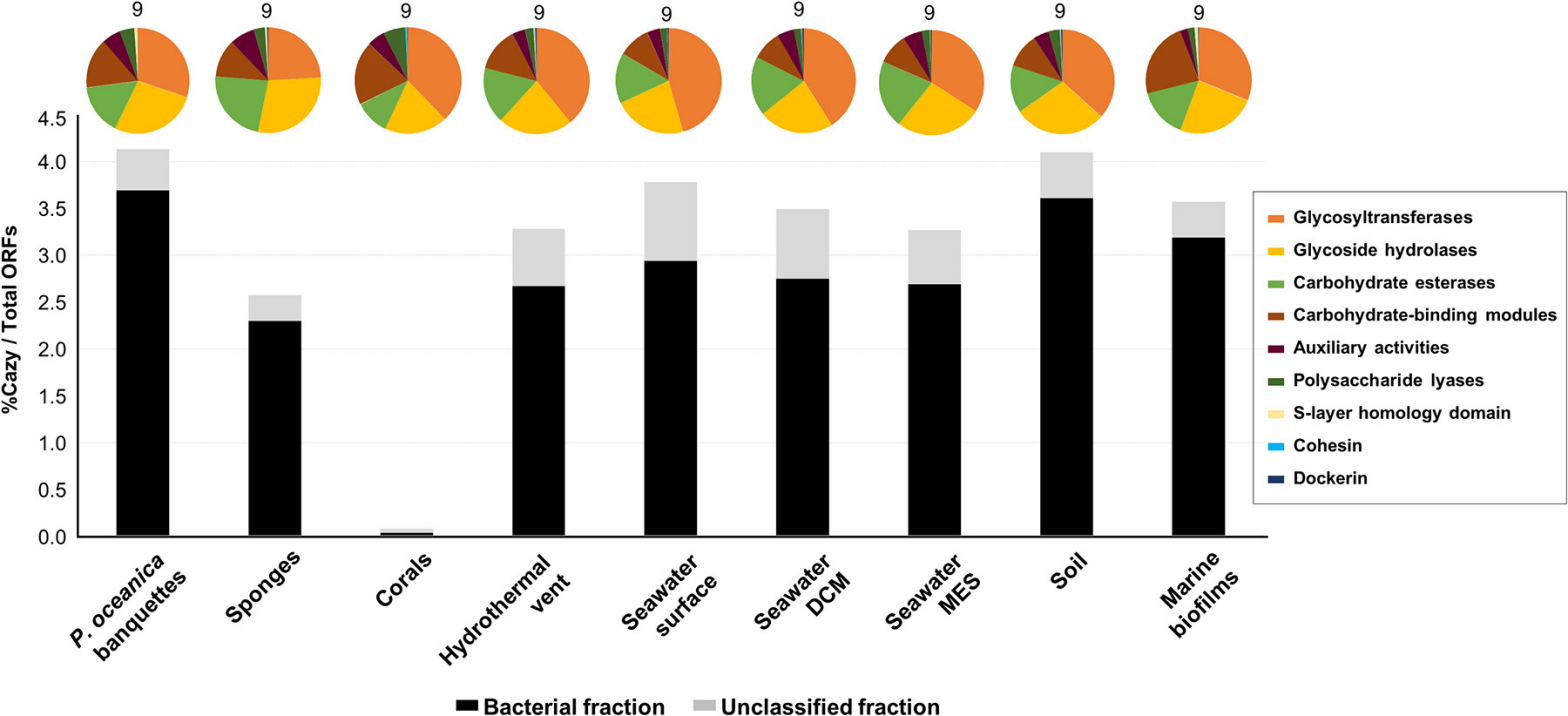
Frequencies of the CAZymes detected using dbCAN in the different environments analyzed in this work. The bar graph shows the number of biosynthetic clusters per number of total ORFs per metagenome. Black indicates the number of CAZymes detected among the bacterial fraction, and gray indicates CAZymes found in the taxonomically unclassified sequences. Pie charts demonstrate the relative contribution of each CAZyme family to the total. The number above each pie chart represents the number of different CAZyme families. DCM, deep chlorophyll maximum; MES, mesopelagic.

**FIG 7:**
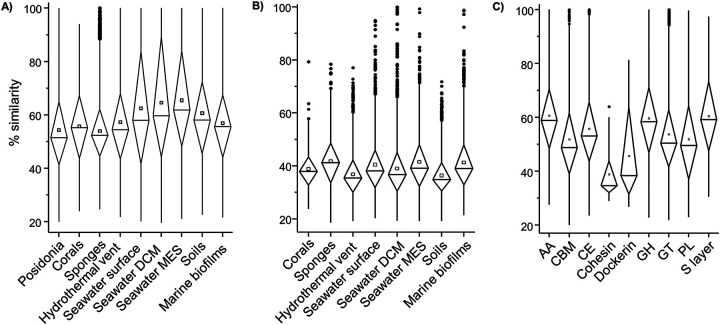
CAZymes and auxiliary enzymes in *Posidonia oceanica* banquettes. (A) Similarities between the CAZyme sequences from the different environments and sequences in the nr database. (B) Similarities between the CAZymes detected in *P. oceanica* and those from the other environments. (C) Similarities between CAZymes from each family detected in *P. oceanica* and sequences in the nr database. DCM, deep chlorophyll maximum; MES, mesopelagic.

Carbohydrate active enzymes (CAZymes) have enormous industrial and biotechnological applications, as they can assemble, modify, and break down oligo- and polysaccharides. A total of 32,891 predicted CAZymes were detected among the >5-kb contigs of *P. oceanica* banquettes; these CAZymes represented 4.13% of the metagenome’s open reading frames (ORFs). Similar numbers were detected in soil (4.09%) and seawater samples (3.26 to 3.77%) (Fig. 6). CAZymes recovered from *P. oceanica* were grouped into 10,480 clusters with above 40% similarity, from which 6,443 were singletons, which suggests the existence of a great diversity of CAZymes in this system. On average, reference sequences from each *P. oceanica* cluster showed 54.39% similarity against the nr database, which is lower than that of the other metagenomes analyzed (Fig. 7A). Only 4,000 of 10,480 CAZyme clusters identified in *P. oceanica* showed similarities over 40% with CAZymes detected in the other environments analyzed. Within this group of homologous CAZymes, the closest to that of *P. oceanica* were detected in seawater and biofilms samples (Fig. 7B).

**FIG 8:**
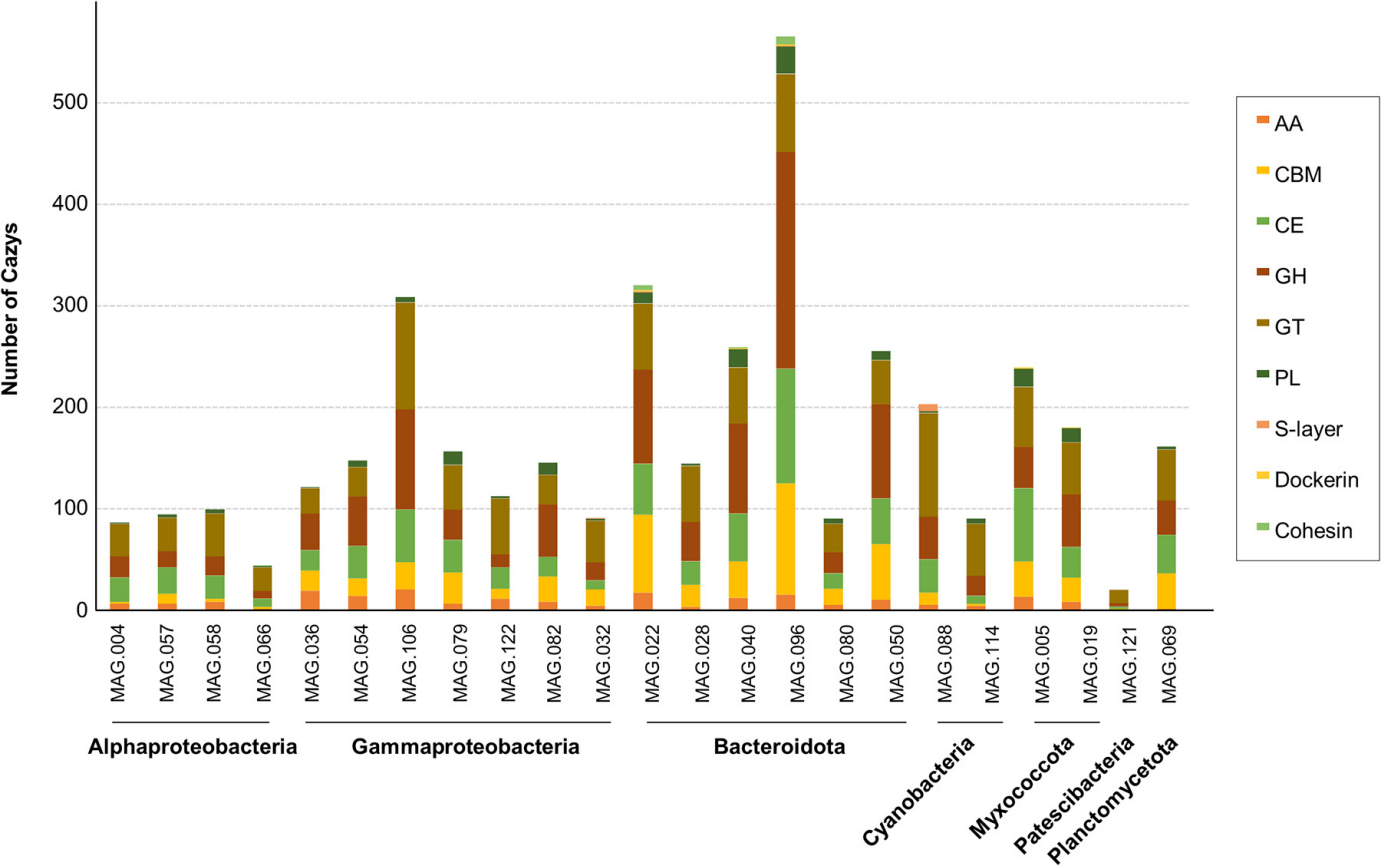
Classification of CAZyme families in the 23 MAGs recovered from *Posidonia oceanica* banquettes.

Among the CAZyme clusters detected in *P. oceanica*, proteins related to dockerins and cohesins showed the lowest identities against the nr database (Fig. 7C). These proteins are keystones for cellulosome assembly, which is a large protein complex found in cellulolytic bacteria that recognizes and degrades plant fiber (63). These protein complexes are usually associated with degradation of plant cell wall polysaccharides and have been mainly described in *Firmicutes* and *Bacteroidetes* (64). Accordingly, in *P. oceanica* banquettes, most proteins assigned to cellulosome modules belonged to *Bacteroidetes* (65.7%). Among the MAGs recovered, only two *Bacteroidetes* MAGs (MAG022 and MAG096) contained cohesin-related genes (Fig. 8). These MAGs also possessed surface layer or S-layer homology (SLH) domains (Fig. 8), which are often used to attach the cellulosome complex to the bacterial cell surface, suggesting the ability of these microorganisms to degrade cellulose. In addition, MAG096, closely related to *Maribacter* spp., encoded the highest observed numbers of putative glycosyl hydrolases (Fig. 8), which are enzymes involved in the degradation of cellulose, hemicellulose, and starch. Previous genomic analyses of species belonging to this genus confirmed their ability to degrade xylan together with alginate and pectin (65). Thus, cells represented by the MAG096 genome may be the cellulosomal bacterium involved in *P. oceanica* banquette degradation. In addition, the five most abundant CAZyme clusters belonged to families related to lignocellulose degradation, including lignocellulose-degrading enzymes and lignocellulose-binding modules (classification of Bredon et al. [66]). This fact confirms that *P. oceanica* banquettes support the presence of lignocellulose-degrading microorganisms, which could be responsible for degrading the plant biomass.

In summary, *P. oceanica* banquettes could provide a unique natural source of microorganisms that produce CAZymes not detected in other environments, mainly proteins assigned to cellulosome modules and lignocellulose-degrading enzymes. These enzymes can be used in many industrial applications, including the fields of cotton processing, paper recycling, and agriculture and for bioenergy production from low-cost lignocellulosic biomass (67).

### Conclusions.

This is the first study of the microbiota diversity in *P. oceanica* banquettes, which showed a high potential for secondary metabolite production, mainly nonribosomal peptides. Importantly, most genes were found in other environments previously examined for this purpose, e.g., soils or seawater. Moreover, *P. oceanica* banquettes harbored several potentially novel bacterial species of putative biotechnological interest; i.e., one MAG belonging to the phylum *Myxoccocota* and another MAG belonging to the genus *Teredinibacter* displayed broad chemical diversity and interesting bioactivities relevant to medical applications.

Furthermore, *P. oceanica* banquettes may also be a good source of new lignocellulose-degrading enzymes with the potential to be used in a wide range of biotechnological processes such as converting lignocellulosic materials into energy fuels. Exploration of alternative fuel resources to face energy shortages is becoming more urgent due to fossil fuel depletion and endeavors to minimize greenhouse gas emissions. Therefore, the development of renewable biofuels has attracted great interest around the world. The discovery of microbial enzymes is especially important to improve the process of converting biomass to biofuels, and metagenomics is a useful method for identifying novel enzymes in environmental samples. Together with lignocellulose-degrading enzymes, novel cellulosome proteins identified in this study represent valuable candidates for further analysis that may, after experimental characterizations, find a future role in biomass conversion applications.

## MATERIALS AND METHODS

### Sampling, DNA extraction, and sequencing.

One sample of *P. oceanica* waste biomass, composed of leaves, roots, and rhizomes, was collected in early September 2016 at Santa Pola Cape (Alicante, Spain) in the Western Mediterranean (38°12′35.4″N, 0°30′27.7″W). *P. oceanica* biomass (10 g) was ground in liquid nitrogen with a sterilized mortar and pestle. Nucleic acids were extracted from the ground biomass using the Power Soil DNA isolation kit (Qiagen) in accordance with the manufacturer’s instructions for maximum yield. DNA library creation and sequencing were performed at Novogen Tech. Co., Ltd. (Beijing, China), on an Illumina HiSeq 4000 platform (2 × 150 bp).

The extracted genomic DNA was also used for PCR amplifications of the V3-V4 region of the 16S rRNA gene by using the universal primers Pro341F (68) and Bact805R (69). The 16S rRNA amplicon sequencing was performed using a 2 × 300 bp paired-end run of Illumina MiSeq Nextera (at Fundació per al Foment de la Investigació Sanitària I Biomédica, FISABIO, Valencia, Spain).

To check the reproducibility of our results on microbial community composition, three additional *P. oceanica* banquette samples were collected from San Juan beach in 2019 and from Rincón de la Zafra and Las Lanzas beaches in 2021 (Alicante, Spain). DNA was extracted and used for PCR amplifications of the V3-V4 region of the 16S rRNA gene as described above.

### 16S metabarcoding analysis.

Downstream bioinformatic analyses of 16S rRNA gene partial sequences were performed using QIIME 1.8.0 (70). Briefly, operational taxonomic units (OTUs) were defined at the level of 99% identity, close to the threshold used to distinguish species (98.7% according to reference 71), followed by taxonomy assignments against the SILVA reference database (version 138) using the UCLUST algorithm (72) and the pick_open_reference_otus.py script.

In addition, 16S rRNA sequences were extracted from the metagenomic data set using the RNAscan software (73) and analyzed as described above for 16S rRNA amplicons. Unidentified reads were screened for the presence of eukaryotic SSU rRNA with Usearch6 (71) against the SILVA-138-SSU database (31) with a cutoff of 97%. Bray-Curtis similarity analyses between different samples at the phylum and class level were done with QIIME 1.8.0 (70).

### Metagenomic analysis.

The sequenced reads were quality trimmed by Trimmomatic (74), and the taxonomic profiling of the entire metagenomic data set was done with Kaiju (75) using the NCBI nr database. Krona viewer was used to display the phylogenetic composition of the *P. oceanica* banquette metagenome (76). Reads were assembled into contigs with MegaHit (77), and their ORFs were extracted using Prodigal (78). The coverage of each contig was calculated through BLASTn comparisons using the BlastTab.seqdepth.pl script from the Enveomics package (79). Since a large proportion of the reads were identified by Kaiju as belonging to *Pseudoalteromonas* but large contigs of that genus were not assembled, we tried to identify its presence in the sample by read recruitment assays against the 428 *Pseudoalteromonas* genomes available on October 2020.

Contigs larger than 5 kb were selected for metagenome binning using two programs, MetaBAT (34) and MaxBin 2.0 (33). The final optimized bins were obtained with DAS Tool 1.0 (35). Genome completeness and the presence of contamination within MAGs were determined with Anvi’o v.2.1.0 (80) and the MIGA platform (using the “NCBI prok” mode) (81). According to the standards suggested by Konstantinidis et al. (82), the quality of the MAGs was considered acceptable when completeness was higher than 80% and contamination was less than 5%. Functional annotation, which also provided taxonomy information, was performed by BLASTp comparisons of the predicted ORFs (from contigs or MAGs) against the NCBI nr database, Pfam (83), COG (84), and TIGRFAM (85) (cutoff E value, 10^−5^). MAGs were manually checked for consistent coverage and taxonomy across contigs.

16S rRNA sequences were extracted from each MAG using the RNAscan software (73) and compared against SILVA-138-SSU (31). In addition to taxonomy information provided during functional annotation, the taxonomy of each MAG was assigned using the MIGA platform (81) and GTDB-tk (86). Both classifications were compared, and incongruences were resolved by phylogenomic reconstructions (see Data Set S1, tab 1, in the supplemental material for taxonomy comparisons). For these reconstructions, a concatenation of the core protein-encoding genes was obtained with PhyloPhlan and aligned with the already built microbial tree of life (containing >3,000 genomes) and other similar genomes to complete the phylogeny (87). The resulting genomic trees were visualized with MegaX (36) (Fig. S1 to S7). The average amino acid identity (AAI) was calculated using CompareM v.0.1.0 (https://github.com/dparks1134/CompareM). Phylogenomic trees were also constructed for those MAGs with an AAI lower than 45% to any other known genome. The relative abundance of each MAG in the *P. oceanica* banquette metagenome was estimated using fragment recruitment analyses carried out by BLASTn comparisons. Only reads that matched with over 95% identity and 70% coverage were considered. Then, the fraction of nucleotides in each sample mapping to the respective MAG was normalized by the length of that MAG and the size of the metagenome. Average nucleotide identity of mapped reads (ANIr) against the reference MAG genome sequence was calculated using all mapped reads with >95% nucleotide identity over 70% of their length, the most common threshold for the distinction of species from sequence data (88). Antibiotic and secondary metabolite biosynthetic genes in MAGs were identified using antiSMASH v.3.0 (89).

### Comparison of putative secondary metabolite production across biomes.

In order to compare the potential production of secondary metabolites by *P. oceanica* banquettes with that of other environments, a total of 60 previously published assembled metagenomes were randomly selected from the Joint Genome Institute (JGI) and NCBI databases and analyzed in parallel (Data Set S1, tab 2). Only contigs larger than 5 kb were used in the subsequent analysis. BLASTn comparisons against the nucleotide (nt) database were used to identify contigs as bacterial, eukaryal, or archaeal (cutoff of >80% identity and a minimum alignment coverage of 40% of the shorter sequence). Secondary metabolite biosynthetic clusters present among the bacterial contigs were identified using antiSMASH v.3.0 (89). This analysis was also performed using the unclassified contigs to expand the detection of novel genes. NaPDoS (http://napdos.ucsd.edu/) (90) was used to predict the condensation (C) and conserved ketoacyl synthase (KS) domains in NRPS and PKS clusters detected by antiSMASH, respectively. To detect redundancy of C and KS domains in each set, protein sequences were clustered using CD-HIT (cutoff, 40% of similarity), and the reference sequence of each cluster was compared to the nr database to check its novelty. The structural class of the product and, when possible, the product structure itself were determined using the NaPDoS database.

### Comparison of carbohydrate active enzymes across biomes.

In order to identify putative enzymes involved in the breakdown, biosynthesis, or modification of carbohydrates, the predicted ORFs from the contigs larger than 5 kb (from all the metagenomes used in this work) were compared against the CAZy database using dbCAN and filtered using suggested cutoffs (91). Annotations of ORFs with significant results were also confirmed by InterProScan (92).

### Data availability.

The raw reads from 16S rRNA metabarcoding and metagenome data sets from *P. oceanica* collected in 2016 were deposited in the NCBI Sequence Read Archive (SRA) database under BioProject accession no. PRJNA662013 and PRJNA662027, respectively. Metagenome-assembled genomes recovered in this study were deposited under BioProject accession no. PRJNA662017. The raw reads from the 16S rRNA metabarcoding data set from *P. oceanica* banquettes collected in 2019 and 2020 were deposited in in the NCBI SRA database under BioProject accession no. PRJNA752987.
