# Supplementary figures and images for: Metagenomics Unveils Posidonia oceanica “Banquettes” as a Potential Source of Novel Bioactive Compounds and Carbohydrate Active Enzymes (CAZymes)
Source: mSystems. 2021 Sep 14;6(5):e00866-21. doi: 10.1128/mSystems.00866-21 (PMC8547425; doi:10.1128/mSystems.00866-21)

Supplementary Figure S8. Portillo et al 2021

A)

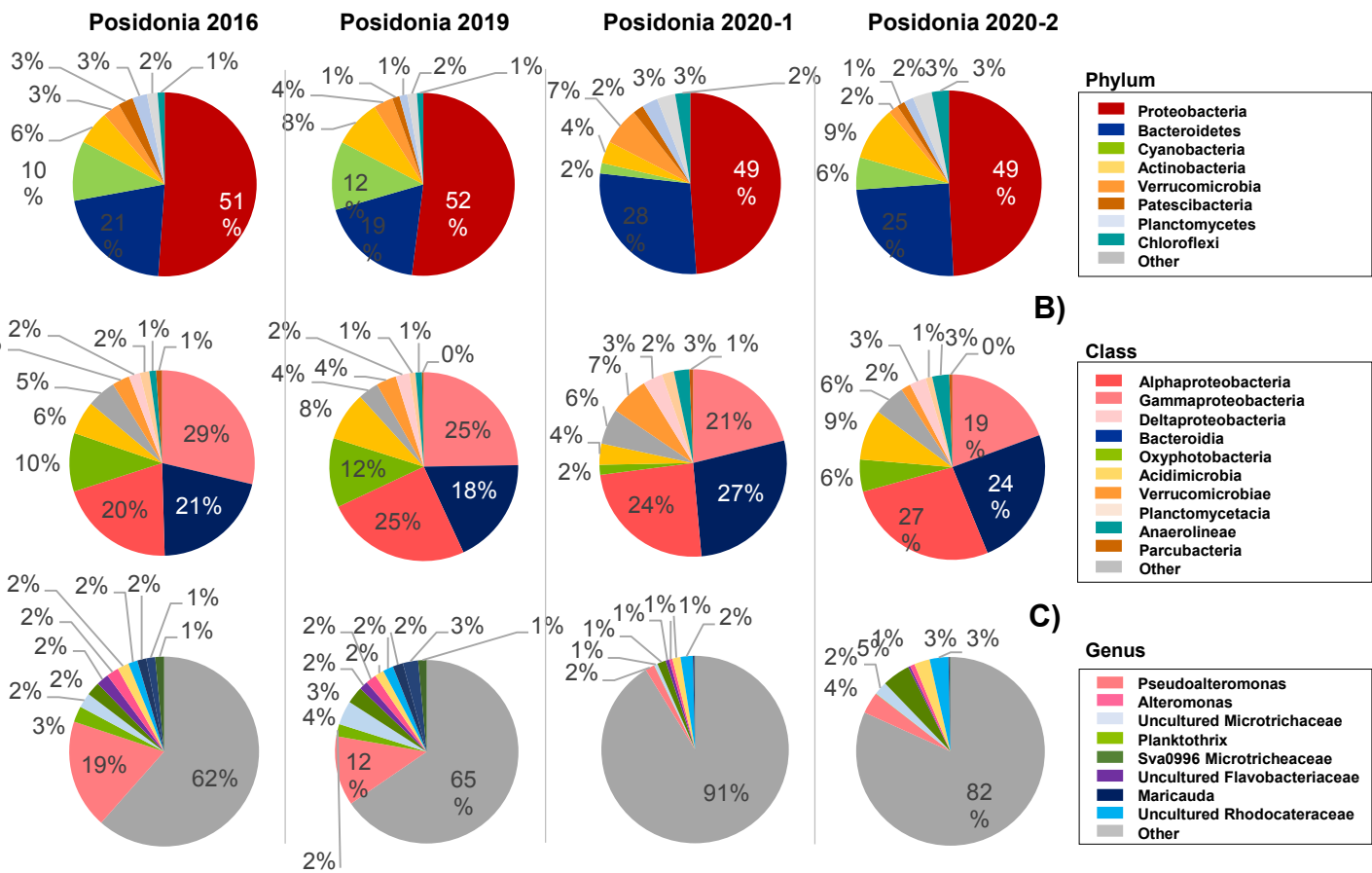

Supplement: FIG S8 [file msystems.00866-21-sf008.pdf]

Size genome (Mb):

2-4

5-7

>8

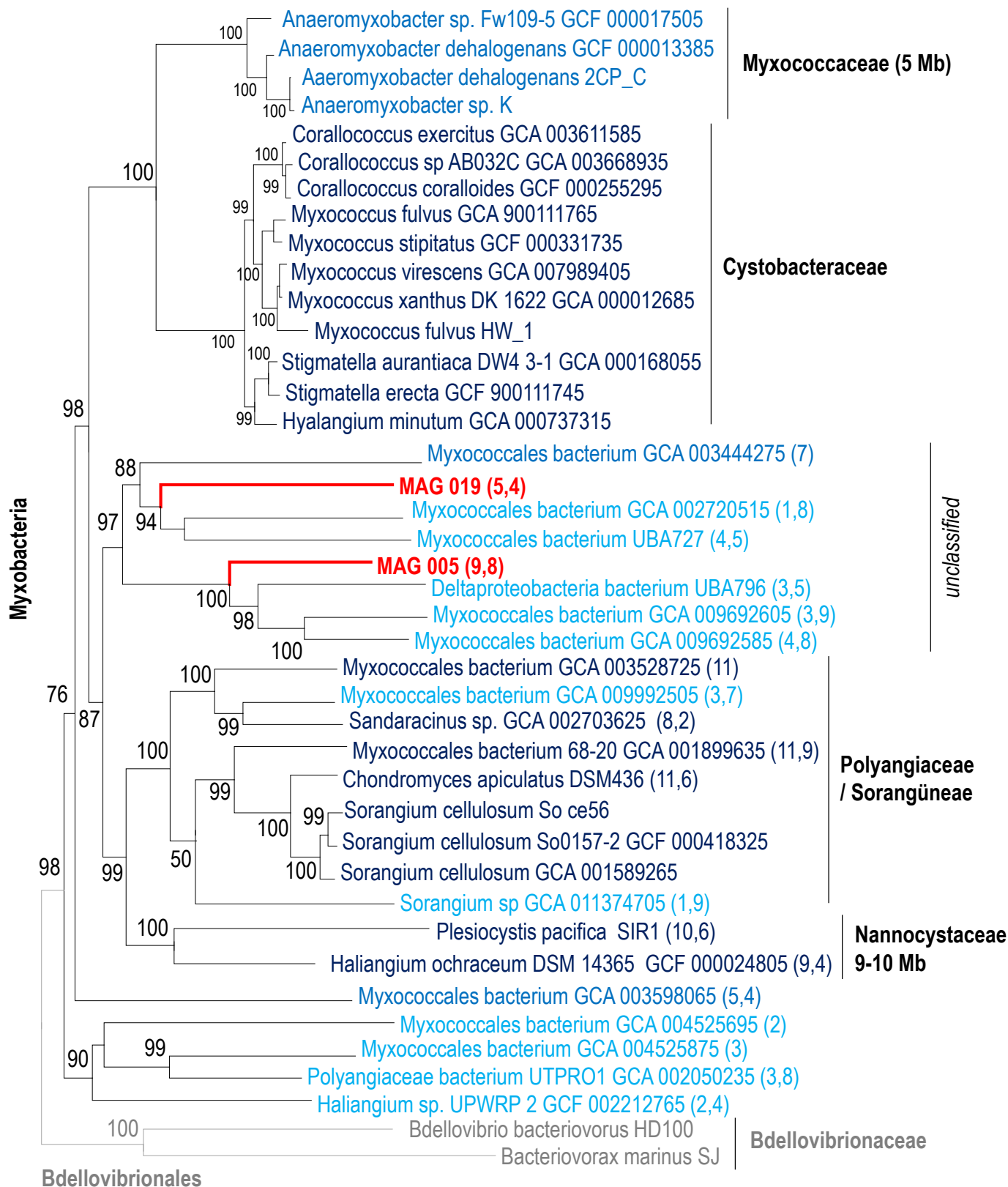

Supplement: FIG S1 [file msystems.00866-21-sf001.pdf]

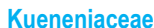

## Phycisphaerae

Supplement: FIG S2 [file msystems.00866-21-sf002.pdf]

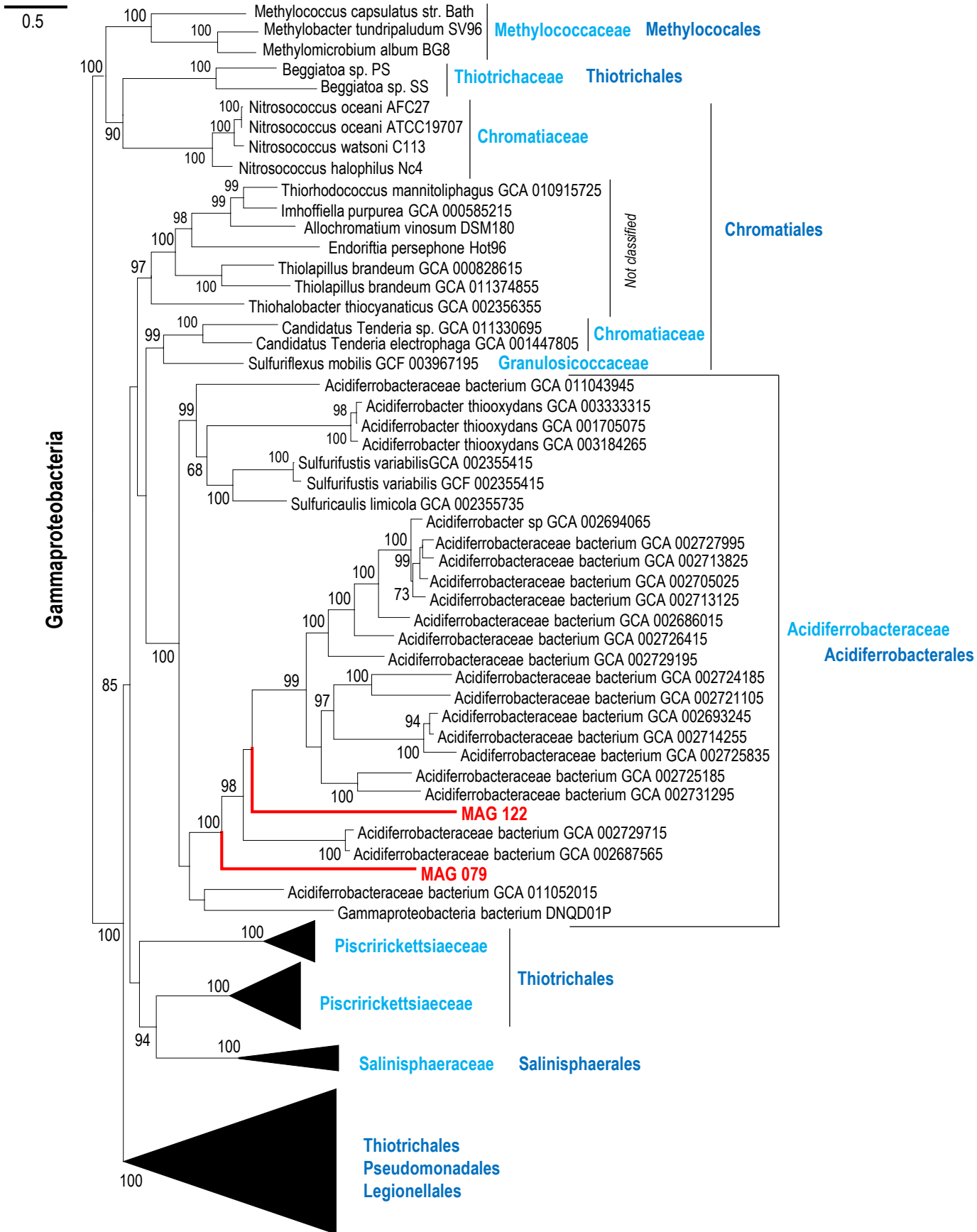

Supplement: FIG S3 [file msystems.00866-21-sf003.pdf]

0.5

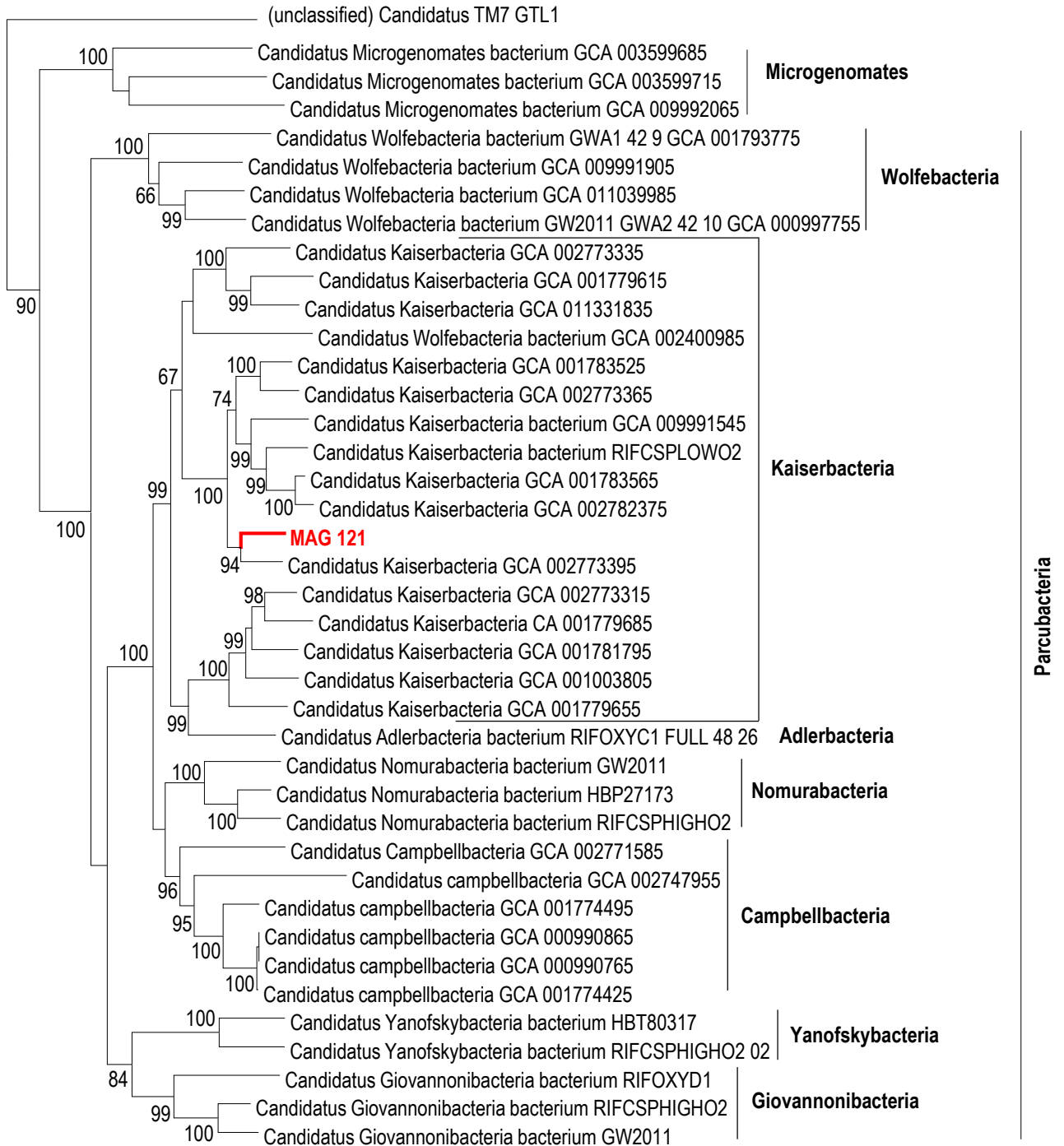

Supplement: FIG S4 [file msystems.00866-21-sf004.pdf]

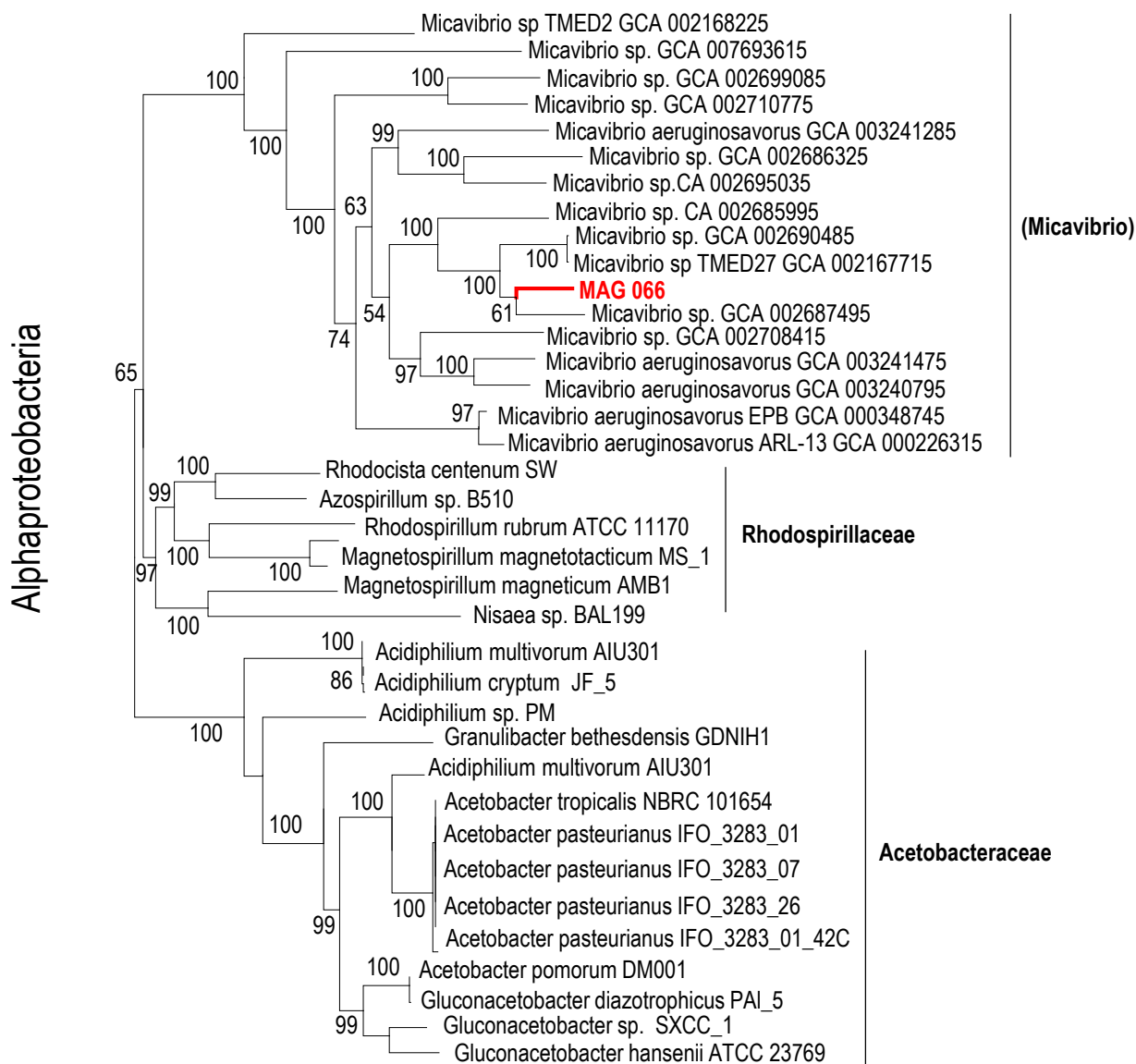

Supplement: FIG S5 [file msystems.00866-21-sf005.pdf]

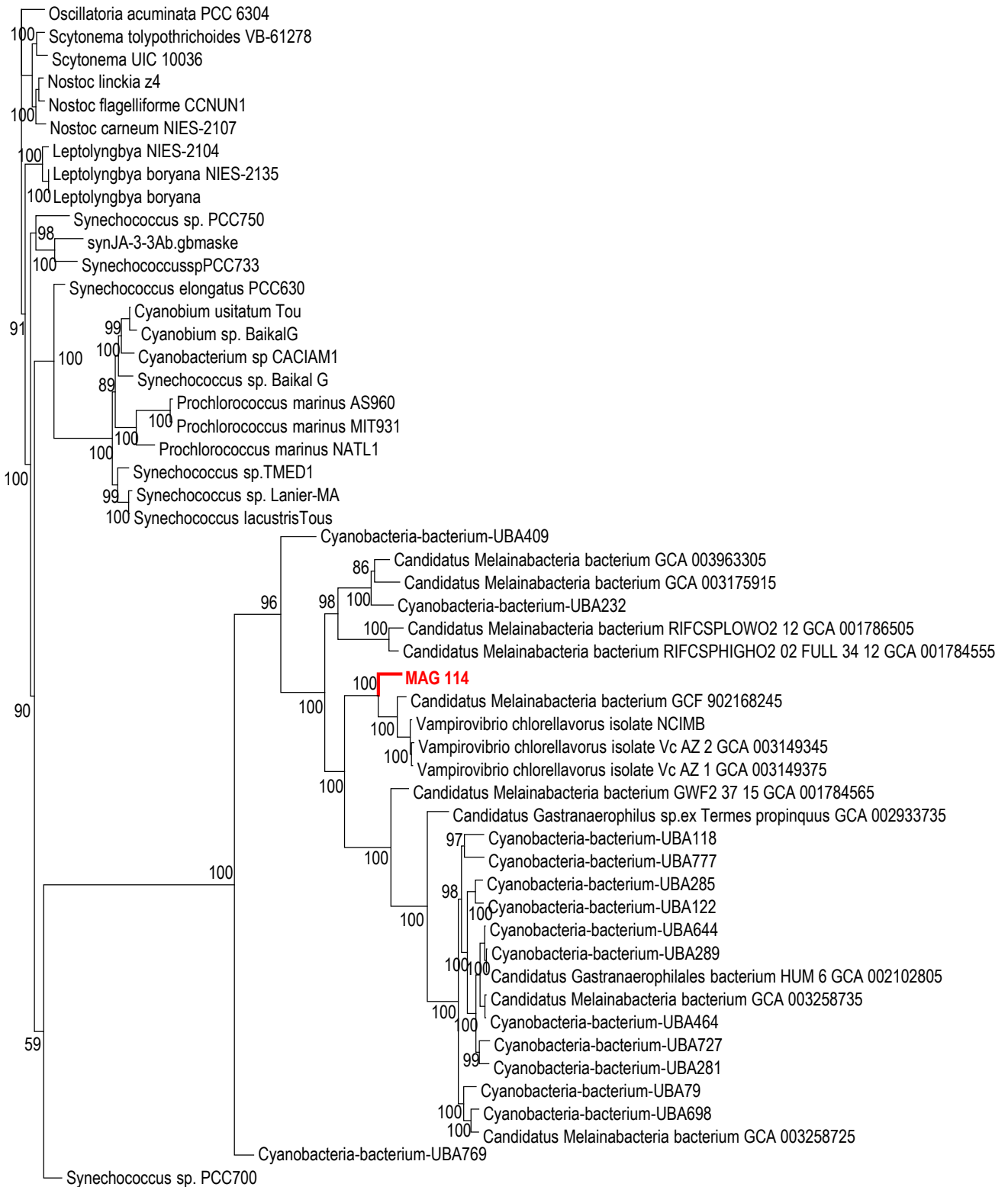

Supplement: FIG S6 [file msystems.00866-21-sf006.pdf]

## Chromatiales (Gammaproteobacteria)

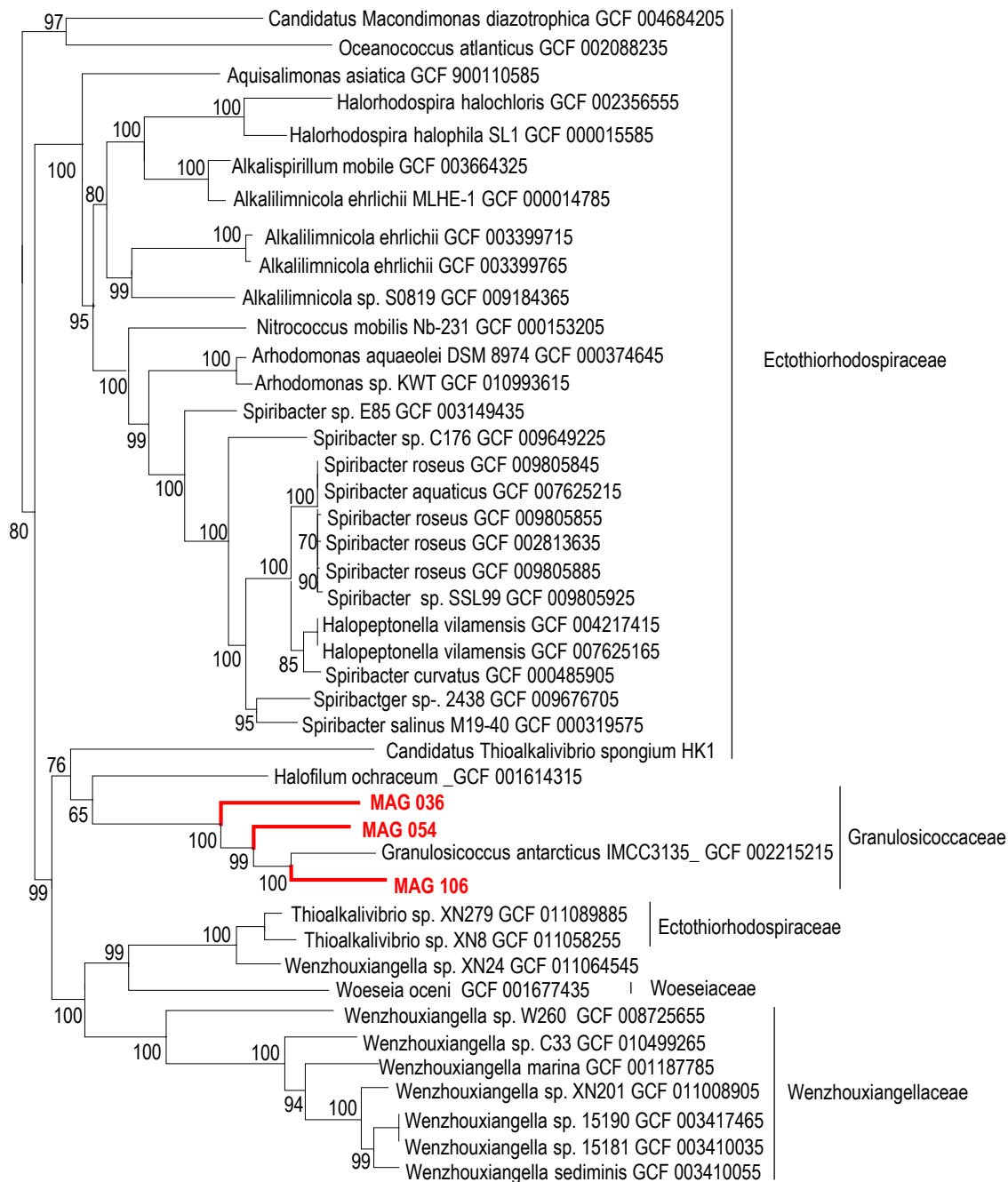

Supplement: FIG S7 [file msystems.00866-21-sf007.pdf]
