# Supplementary material for: Metagenomics Unveils Posidonia oceanica “Banquettes” as a Potential Source of Novel Bioactive Compounds and Carbohydrate Active Enzymes (CAZymes)
Source: mSystems. 2021 Sep 14;6(5):e00866-21. doi: 10.1128/mSystems.00866-21 (PMC8547425; doi:10.1128/mSystems.00866-21)
Supplement: FIG S9 [file msystems.00866-21-sf009.pdf]

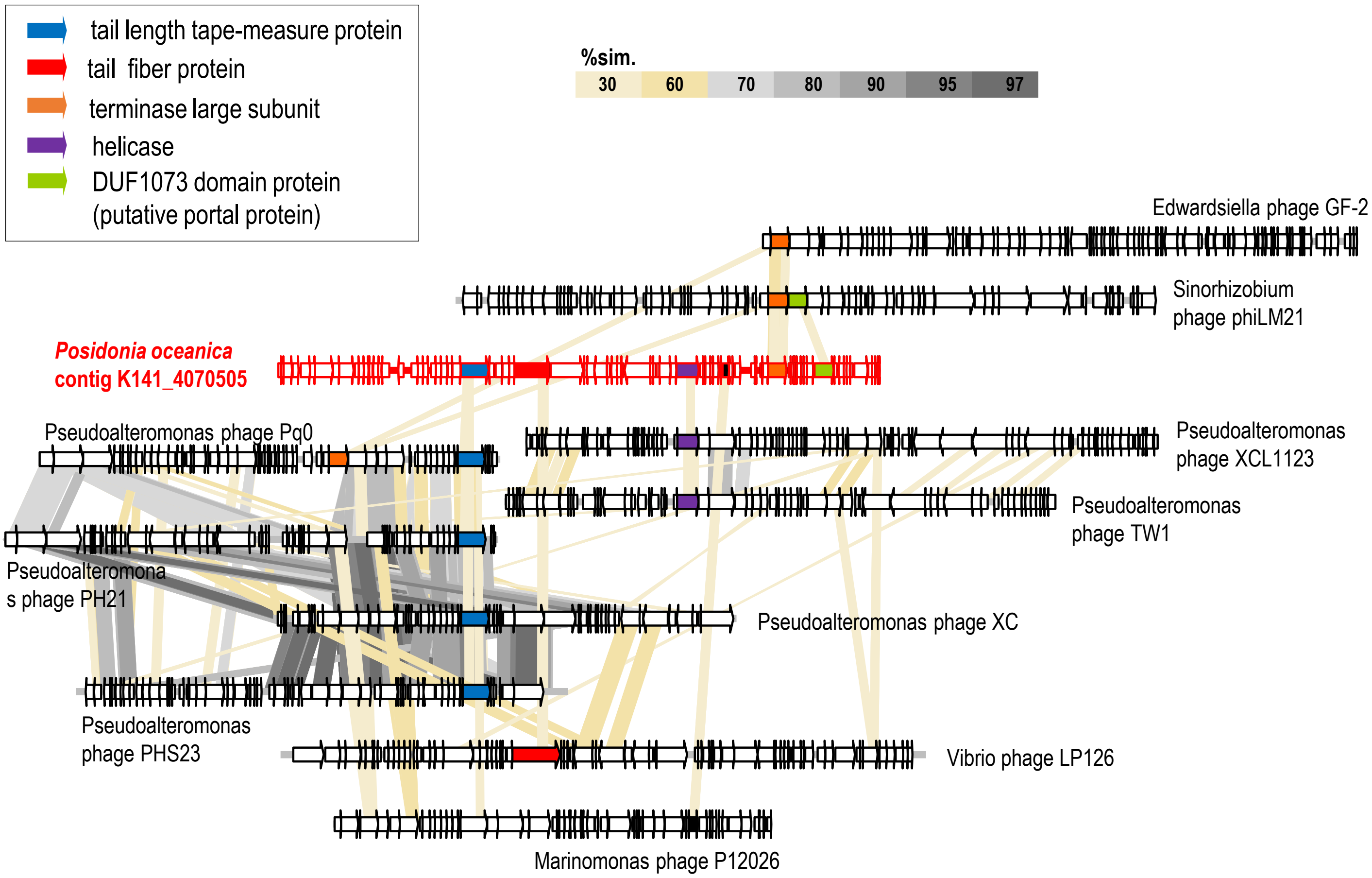

tail length tape-measure protein
tail fiber protein
terminase large subunit
helicase
DUF1073 domain protein
(putative portal protein)
%sim.
30
60
70
80
90
95
97
Edwardsiella phage GF-2
Sinorhizobium
phage phiLM21
Posidonia oceanica
contig K141_4070505
Pseudoalteromonas phage Pq0
Pseudoalteromonas
phage XCL1123
Pseudoalteromonas
phage TW1
Pseudoalteromona
s phage PH21
Pseudoalteromonas phage XC
Pseudoalteromonas
phage PHS23
Vibrio phage LP126
Marinomonas phage P12026
